# Physiological and Recovery Responses to Dietary Polyphenols in the Context of Exercise: Relevance for Muscle Aging and Sarcopenia

**DOI:** 10.3390/nu18050788

**Published:** 2026-02-27

**Authors:** Vince Fazekas-Pongor, Dávid Major, János Tamás Varga, Andrea Lehoczki, Péter Varga, Tamás Jarecsny, Ágnes Lipécz, Tamás Csípő, Ágnes Szappanos, Attila Matiscsák, Mónika Fekete

**Affiliations:** 1 Institute of Preventive Medicine and Public Health, Faculty of Medicine, Semmelweis University, 1085 Budapest, Hungary; pongor.vince@semmelweis.hu (V.F.-P.); major.david@semmelweis.hu (D.M.); ceglediandi@freemail.hu (A.L.); varga.peter@semmelweis.hu (P.V.); lipecz.agnes@semmelweis.hu (Á.L.); csipo.tamas@semmelweis.hu (T.C.); 2 Health Sciences Division, Doctoral College, Semmelweis University, 1085 Budapest, Hungary; 3 Fodor Center for Prevention and Healthy Aging, Semmelweis University, 1085 Budapest, Hungary; 4 Department of Pulmonology, Semmelweis University, 1083 Budapest, Hungary; varga.janos.tamas@semmelweis.hu; 5 Department of Neurology and Stroke, Saint John’s Central Hospital of North Buda, 1125 Budapest, Hungary; jarecsny.tamas@janoskorhaz.hu; 6 Heart and Vascular Center, Semmelweis University, 1122 Budapest, Hungary; drszappanos@gmail.com; 7 S-CAPE Cognitive and Health Prevention Research Group, Faculty of Health Sciences, Semmelweis University, 1088 Budapest, Hungary; matiscsak.attila@semmelweis.hu; 8 Department of Social Sciences, Faculty of Health Sciences, Semmelweis University, 1088 Budapest, Hungary

**Keywords:** sarcopenia, polyphenols, resistance training, aging, skeletal muscle, anabolic resistance, mitochondrial dysfunction, inflammation, redox signaling

## Abstract

Introduction: The biological effects of dietary polyphenols have gained increasing attention due to their roles in regulating oxidative stress, inflammatory processes, and mitochondrial function. Human studies suggest that polyphenol intake may support aspects of post-exercise recovery, neuromuscular function, and selected aspects of physical performance. However, most investigations have been conducted in young or metabolically healthy populations, limiting direct clinical translation to older adults. Objective: This narrative review aims to synthesize current mechanistic and human evidence on the physiological and recovery-related effects of dietary polyphenols in the context of exercise adaptation and skeletal muscle function, and to examine their potential relevance to muscle aging and sarcopenia. Methods: A structured, non-systematic literature search was conducted to integrate findings from human intervention trials, preclinical studies, and mechanistic research addressing polyphenols, exercise adaptation, muscle recovery, and muscle aging. Evidence was synthesized narratively with emphasis on shared physiological pathways and functional outcomes. Results: Human intervention studies suggest that polyphenol intake may attenuate biomarkers of exercise-induced muscle damage, modulate inflammatory responses, and accelerate recovery of muscle strength and functional performance. Mechanistic evidence supports the involvement of redox homeostasis, mitochondrial regulation, and inflammatory signaling as central mediators of these effects. While clinical data in older populations remain limited, converging evidence suggests biological overlap between recovery-related pathways and mechanisms implicated in age-related muscle decline. Conclusions: Current evidence is consistent with a biologically plausible role for polyphenols in modulating exercise-related physiological and recovery processes. By aligning recovery-focused evidence with pathways central to muscle aging, this review proposes a translational framework that may inform the design of future targeted clinical trials in older and clinical populations.

## 1. Introduction

Skeletal muscle adaptation to exercise and the subsequent recovery process are key determinants of physical performance, functional capacity, and long-term muscle health [1]. Intense or unaccustomed physical activity can induce transient muscle damage, inflammatory responses, oxidative stress, and metabolic alterations, the magnitude and time course of which influence recovery kinetics and the quality of adaptive responses [2]. These processes are relevant not only to sports physiology but also to understanding the mechanisms underlying muscle aging and functional decline [3]. In recent years, increasing attention has been directed toward nutritional factors capable of modulating physiological responses to exercise [4,5,6,7,8,9,10,11,12,13]. Among these, dietary polyphenols have attracted considerable interest, as experimental and human studies suggest that they may influence inflammatory pathways, redox homeostasis, and mitochondrial function [14,15,16,17,18]. These mechanisms play central roles in muscle recovery and adaptation [18,19,20,21,22].

The biological effects of polyphenols extend beyond their traditional classification as antioxidants [23,24,25,26]. Growing evidence indicates that these bioactive compounds can modulate intracellular signaling pathways and induce adaptive cellular responses, including mechanisms consistent with mitohormesis, thereby enhancing cellular stress resistance and metabolic flexibility [27]. However, it is important to note that a substantial proportion of human intervention studies have been conducted in young, healthy, or recreationally active populations, and therefore, the direct applicability of these findings to clinical or older populations remains limited.

Notably, several physiological mechanisms involved in exercise recovery overlap with processes implicated in muscle aging, including chronic low-grade inflammation, oxidative stress, mitochondrial dysfunction, and anabolic resistance [28]. This overlap raises the possibility that factors influencing recovery responses may have relevance for aging muscle, although direct clinical evidence supporting this translational link is still limited.

Resistance training remains one of the most effective strategies to maintain or improve muscle function, yet considerable inter-individual variability in adaptive responses has been consistently observed, particularly in older adults [29,30,31,32,33,34,35,36]. Differences in recovery capacity, inflammatory regulation, and metabolic responses are likely contributors to this variability, further supporting interest in nutritional and lifestyle factors that may modulate these processes [37,38,39].

The present narrative review synthesizes and critically interprets evidence on the physiological and recovery-related effects of dietary polyphenols in the context of exercise and skeletal muscle function, examining their potential translational relevance for muscle aging and sarcopenia. The emphasis is placed on mechanistic and recovery-related pathways, with clinical implications considered from a translational, hypothesis-generating perspective rather than as established therapeutic recommendations.

## 2. Methods

### 2.1. Literature Search Strategy

This narrative review was conducted using a structured but non-systematic literature search strategy aimed at integrating mechanistic and clinical evidence on physiological and recovery-related responses to dietary polyphenols in the context of exercise, and discussing their potential translational relevance for muscle aging. A comprehensive search was performed in PubMed/MEDLINE, Scopus, and Web of Science to identify studies investigating polyphenols, exercise, skeletal muscle adaptation, and recovery processes. The search covered articles published between January 2000 and March 2025 and was limited to English-language publications. Search terms included combinations of “polyphenols”, “exercise”, “exercise recovery”, “muscle damage”, “skeletal muscle”, “resistance training”, “mitochondrial function”, “oxidative stress”, “inflammation”, “muscle protein synthesis”, “aging muscle”, and “sarcopenia”. The initial search identified approximately 5200 records. Additional studies were identified through manual screening of reference lists. After duplicate removal, titles and abstracts were screened, followed by full-text evaluation of potentially relevant studies. Approximately 120 full texts were assessed, and about 60 studies were included in the qualitative synthesis.

Priority was given to studies that conducted the following:

- investigated physiological or recovery responses to exercise;
- included human participants;
- provided mechanistic insights with potential relevance for muscle aging.

The aim of this structured search was to improve transparency while maintaining the integrative nature of a narrative review.

### 2.2. Eligibility Criteria

Studies were considered eligible if they met at least one of the following criteria:

- human randomized controlled trials, observational studies, or intervention studies examining physiological responses, recovery, or muscle function in relation to polyphenol intake;
- preclinical studies providing mechanistic insights into skeletal muscle adaptation;
- systematic reviews addressing exercise- or nutrition-related effects on muscle function.

Studies focusing specifically on sarcopenia were considered in the context of translational interpretation but were not required for inclusion. Case reports, conference abstracts, non-peer-reviewed publications, and studies not directly related to skeletal muscle physiology or exercise responses were excluded.

### 2.3. Study Selection and Data Extraction

Titles and abstracts were screened, followed by full-text assessment. Data were extracted on study design, population characteristics, intervention type and duration, and outcomes related to muscle function, recovery, and mechanistic endpoints. Data synthesis focused on qualitative integration rather than quantitative pooling.

### 2.4. Quality Assessment and Risk of Bias

Due to heterogeneity in study designs and outcomes, formal meta-analysis was not performed. Study quality and potential sources of bias were evaluated qualitatively, with attention to study design, sample size, intervention fidelity, and outcome assessment. Particular emphasis was placed on evaluating the translational relevance of findings for aging muscle and clinical populations.

### 2.5. Data Synthesis

Findings were synthesized narratively. Mechanistic and clinical evidence were integrated to identify converging biological pathways involved in exercise recovery and skeletal muscle adaptation. Clinical implications were interpreted cautiously and primarily from a hypothesis-generating perspective.

## 3. Physiological Mechanisms of Muscle Aging Relevant to Exercise Adaptation and Recovery

Age-related alterations in skeletal muscle physiology influence not only the development of sarcopenia but also responses to exercise and post-exercise recovery. Understanding these mechanisms provides an important framework for interpreting how nutritional factors, including dietary polyphenols, may modulate exercise-induced adaptations.

### 3.1. Anabolic Resistance and Impaired Muscle Protein Synthesis

One of the central mechanisms underlying age-related loss of skeletal muscle mass is anabolic resistance, defined as a reduced responsiveness of muscle protein synthesis to both nutritional and mechanical stimuli [40,41]. In older adults, skeletal muscle exhibits a blunted anabolic response to dietary protein intake and to mechanical loading induced by resistance exercise [37]. Over time, this impaired responsiveness contributes to a chronic negative muscle protein balance and progressive muscle wasting [42].

A key regulator of anabolic signaling in skeletal muscle is the mechanistic target of rapamycin complex 1 (mTORC1), which integrates amino acid availability, growth factor signaling, and mechanical stimuli to promote muscle protein synthesis [43]. With advancing age, the activity of the insulin-like growth factor 1 (IGF-1)–phosphatidylinositol 3-kinase (PI3K)–protein kinase B (Akt)–mechanistic target of rapamycin complex 1 signaling cascade is attenuated [44,45]. This impairment is driven by alterations at both receptor and post-receptor levels, as well as by inhibitory influences from inflammatory mediators and metabolic stress.

A hallmark feature of anabolic resistance is reduced sensitivity to leucine, a potent activator of muscle protein synthesis [46]. Consequently, higher protein or essential amino acid intake is required in older individuals to achieve anabolic responses comparable to those observed in younger adults [47]. This diminished nutrient sensitivity limits the effectiveness of nutrition alone and has direct implications for exercise adaptation, as impaired anabolic signaling may constrain recovery and training responsiveness [48,49].

### 3.2. Chronic Low-Grade Inflammation and Oxidative Stress

Aging is accompanied by a state of chronic low-grade systemic inflammation, often referred to as inflammaging, which represents a major contributor to the pathophysiology of sarcopenia [50]. Persistently elevated circulating levels of pro-inflammatory cytokines, including interleukin 6 (IL-6) and tumor necrosis factor alpha (TNF-α), adversely affect muscle protein metabolism by suppressing anabolic signaling and promoting catabolic pathways [51].

The nuclear factor kappa-light-chain-enhancer of activated B cells (NF-κB) signaling pathway plays a central role in mediating inflammation-induced muscle dysfunction [52]. Sustained activation of this pathway enhances the expression of genes involved in muscle protein degradation and impairs myogenic regeneration. However, it is important to emphasize that inflammatory and oxidative processes are not inherently pathological. At physiological levels, these signals are essential for the regulation of tissue remodeling and adaptation, particularly in response to physical exercise [53,54].

Similarly, reactive oxygen species (ROS) should not be viewed solely as damaging by-products of metabolism [55]. Moderate and transient increases in reactive oxygen species act as critical signaling molecules that regulate exercise-induced adaptations, mitochondrial biogenesis, and muscle regeneration [56]. In the context of sarcopenia, pathology arises not from the presence of reactive oxygen species per se, but from disrupted redox homeostasis and dysregulated redox-sensitive signaling [57]. Because inflammatory and redox-sensitive pathways play a central role in both muscle recovery and aging-related muscle decline, they represent key biological nodes through which dietary polyphenols may modulate recovery and aging-related muscle processes.

### 3.3. Mitochondrial Dysfunction and Impaired Metabolic Flexibility

Sarcopenia is frequently associated with mitochondrial dysfunction, characterized by reduced mitochondrial content, impaired oxidative capacity, and decreased energy production efficiency [58]. Aging skeletal muscle exhibits a decline in mitochondrial biogenesis, accompanied by reduced activity of key regulatory factors that govern mitochondrial turnover and function [59]. These alterations contribute to diminished muscular endurance, impaired recovery, and increased susceptibility to fatigue.

In parallel, aging is associated with a loss of metabolic flexibility, defined as the ability of skeletal muscle to efficiently switch between lipid and glucose oxidation in response to physiological demands [60]. Mitochondrial dysfunction and metabolic inflexibility reinforce one another, exacerbating disturbances in muscle energy metabolism and accelerating functional decline [61].

Importantly, mitochondria-derived reactive oxygen species serve essential signaling roles in the regulation of cellular adaptation and homeostasis [62]. In healthy muscle, these signals support mitochondrial remodeling and stress resilience [63]. In sarcopenia, however, excessive or poorly regulated reactive oxygen species production disrupts these signaling pathways, contributing to impaired adaptation and progressive muscle dysfunction [64]. Impaired mitochondrial function contributes to sarcopenia and compromises exercise tolerance, recovery kinetics, and metabolic adaptation, positioning mitochondrial pathways as a central mechanistic link between exercise physiology and nutritional interventions.

## 4. Resistance Training as a Core Intervention Against Sarcopenia

The evidence summarized in this section derives from randomized controlled trials, longitudinal training studies, and mechanistic investigations examining molecular adaptations to resistance exercise, identified through the structured literature search described in the Section 2.

### 4.1. Effects of Resistance Training on Muscle Mass and Strength

Resistance training is widely recognized as one of the most effective evidence-based interventions for the prevention and treatment of sarcopenia [65]. Findings from randomized controlled trials and meta-analyses consistently demonstrate that resistance exercise substantially improves muscle strength in older adults, while inducing modest but clinically meaningful increases in skeletal muscle mass [66]. In addition, resistance training has been shown to enhance physical function and performance, thereby supporting independence and quality of life in aging populations [67].

The adaptive response to resistance training follows a dose–response pattern influenced by training intensity, volume, frequency, and duration. In older individuals, moderate- to high-intensity progressive resistance training appears to be most effective for increasing muscle strength, whereas gains in muscle mass are often smaller and characterized by considerable interindividual variability [68]. This observation suggests that improvements in strength are not solely attributable to increases in muscle size but are also driven by neural adaptations and improvements in neuromuscular coordination [69].

### 4.2. Molecular Adaptations Induced by Resistance Training

Resistance training elicits a broad range of molecular and cellular adaptations within skeletal muscle that are essential for regulating muscle protein metabolism and tissue remodeling [4]. Mechanical loading activates anabolic signaling pathways, most notably those involving the mechanistic target of rapamycin complex 1, which plays a central role in stimulating muscle protein synthesis and promoting muscle fiber hypertrophy [70].

In addition to anabolic signaling, resistance training promotes the activation and proliferation of muscle satellite cells, a population of myogenic precursor cells that contribute to muscle regeneration and long-term maintenance of muscle tissue [71]. Although satellite cell function and responsiveness decline with age, resistance exercise remains a potent stimulus capable of inducing adaptive responses even in older skeletal muscle [72].

While resistance training is primarily associated with improvements in muscle strength and mass, accumulating evidence indicates that it also influences mitochondrial function [73,74]. Resistance exercise can enhance mitochondrial quality, improve mitochondrial dynamics, and increase oxidative capacity, particularly when training programs are progressive and of sufficient volume [75]. These mitochondrial adaptations may contribute to improved metabolic efficiency and stress resilience in aging skeletal muscle.

### 4.3. Limitations and Heterogeneity of Training Responses

Despite the well-established benefits of resistance training, considerable heterogeneity exists in individual responses among older adults. A subset of individuals demonstrates minimal or no measurable improvements in muscle mass or strength despite adherence to structured training programs, a phenomenon often described as non-responsiveness [76]. This variability in training outcomes is likely driven by a complex interplay of factors, including anabolic resistance, chronic low-grade inflammation, mitochondrial dysfunction, genetic predisposition, and baseline physical function [77].

Adherence represents an additional and critical limitation of resistance training interventions in older populations [78]. Physical limitations, comorbidities, motivational factors, and environmental barriers frequently reduce long-term participation in exercise programs [79]. Poor adherence not only diminishes the effective training dose but also limits the development and maintenance of adaptive responses [78].

These constraints suggest that resistance training alone may not fully address the multifactorial nature of sarcopenia in all individuals. This has stimulated interest in complementary, multimodal strategies—particularly targeted nutritional interventions—that may support recovery, modulate inflammatory and redox pathways, and optimize training responsiveness. Among these, dietary polyphenols have emerged as candidates of growing interest [80].

## 5. Polyphenol-Rich Foods and Their Biological Effects on Skeletal Muscle

Dietary polyphenols constitute a structurally diverse class of bioactive compounds that modulate multiple physiological pathways relevant to skeletal muscle function, exercise adaptation, and recovery. Given substantial differences in bioavailability, molecular targets, and effective dosing among individual compounds, this section does not seek to compare specific polyphenols but rather to delineate converging mechanistic pathways through which polyphenol-rich foods may influence skeletal muscle physiology. These mechanisms are examined primarily within the context of exercise-induced stress and recovery, with their relevance to muscle aging and sarcopenia addressed from a translational perspective.

### 5.1. Classification and Dietary Sources of Polyphenols

Polyphenols represent a large and heterogeneous group of bioactive plant-derived compounds characterized by the presence of multiple phenolic structures [81]. Based on their chemical structure, dietary polyphenols are commonly classified into major subclasses, including flavonoids, stilbenes, and phenolic acids, each of which encompasses numerous individual compounds with distinct biological activities [27,81,82].

Flavonoids constitute the most abundant class of dietary polyphenols and include subclasses such as flavonols, flavanols, flavones, flavanones, isoflavones, and anthocyanins [83]. These compounds are widely distributed in fruits, vegetables, tea, cocoa, and legumes. Stilbenes, of which resveratrol is the most extensively studied representative, are found primarily in grapes, red wine, and certain berries. Phenolic acids, including hydroxybenzoic and hydroxycinnamic acids, are common components of coffee, whole grains, fruits, and vegetables [84,85].

Importantly, polyphenols are typically consumed as complex mixtures within whole foods rather than as isolated compounds. Whole-food sources provide polyphenols in combination with dietary fiber, micronutrients, and other bioactive components that may influence absorption, metabolism, and biological activity [86]. In contrast, isolated polyphenol supplements often deliver higher doses of single compounds but lack the food matrix that may modulate bioavailability and physiological responses [87]. This distinction is particularly relevant when interpreting experimental findings and translating them into dietary recommendations [88]. Table 1 summarizes the major classes of dietary polyphenols, their primary food sources, and key considerations regarding bioavailability and skeletal muscle relevance.

### 5.2. Anti-Inflammatory and Redox-Modulating Effects

The evidence summarized in this section is drawn primarily from mechanistic investigations and selected human studies examining inflammatory and redox-regulated pathways involved in exercise-induced muscle stress and recovery. Among the most extensively characterized biological properties of polyphenols is their capacity to modulate these interconnected signaling networks. Rather than functioning merely as direct antioxidants, polyphenols influence intracellular cascades that coordinate inflammatory responses, redox homeostasis, and adaptive cellular processes [89,90].

Polyphenols have been shown to attenuate the activation of the nuclear factor kappa-light-chain-enhancer of activated B cells (NF-κB) pathway, a central regulator of pro-inflammatory gene expression [91]. Through modulation of this pathway, polyphenols may reduce the production of pro-inflammatory cytokines and mitigate chronic low-grade inflammation, a key contributor to age-related skeletal muscle dysfunction [91,92].

In parallel, polyphenols activate endogenous antioxidant defense systems through the nuclear factor erythroid 2-related factor 2 (Nrf2) pathway [93]. Activation of nuclear factor erythroid 2-related factor 2 promotes the expression of genes involved in cellular detoxification, redox balance, and mitochondrial protection [94]. This indirect regulation of redox homeostasis supports cellular resilience without suppressing physiologically important reactive oxygen species signaling [94].

These effects are consistent with the concept of hormesis, whereby low to moderate levels of biological stress induce adaptive responses that enhance cellular function and stress resistance. In this context, polyphenols act as mild stressors that stimulate adaptive signaling rather than as simple radical scavengers, an important distinction when considering their role in skeletal muscle health [95,96]. These signaling pathways are also implicated in muscle aging, which provides a biologically plausible rationale for considering the translational relevance of these findings to sarcopenia, although direct clinical evidence remains limited [95,96].

### 5.3. Effects on Mitochondrial Function and Muscle Metabolism

The evidence summarized in this section derives from preclinical mechanistic studies and a limited number of human investigations exploring mitochondrial regulation and metabolic adaptation in skeletal muscle. Polyphenols may exert pleiotropic effects on mitochondrial dynamics and muscle metabolism through modulation of key energy-sensing and transcriptional networks. Central to these mechanisms is activation of sirtuin 1 (SIRT1) and adenosine monophosphate-activated protein kinase (AMPK), pivotal regulators of cellular energy homeostasis and metabolic flexibility [97].

Activation of sirtuin 1 and adenosine monophosphate-activated protein kinase converges on peroxisome proliferator-activated receptor gamma coactivator 1 alpha (PGC-1α), a master regulator of mitochondrial biogenesis and oxidative metabolism [98]. Through this signaling network, polyphenols have been shown in preclinical models to enhance mitochondrial content, improve mitochondrial efficiency, and promote oxidative capacity in skeletal muscle [97].

Much of the mechanistic evidence supporting these effects originates from preclinical models, including rodent and cellular studies, in which polyphenols such as resveratrol and flavonoids robustly activate mitochondrial biogenesis and improve metabolic flexibility [97,99,100,101]. In contrast, human studies remain more limited and yield heterogeneous results, likely reflecting differences in polyphenol dose, bioavailability, intervention duration, and baseline metabolic status. Nevertheless, emerging human data suggest that polyphenol-rich dietary patterns may support mitochondrial function, particularly in aging or metabolically compromised populations.

### 5.4. Polyphenols and Muscle Protein Turnover

Beyond their effects on inflammation and mitochondrial metabolism, polyphenols may also influence skeletal muscle protein turnover by modulating anabolic and catabolic signaling pathways [102,103]. Experimental studies indicate that polyphenols can interact with the mTOR pathway, a central regulator of muscle protein synthesis [103,104,105]. Rather than chronically activating this pathway, polyphenols appear to fine-tune its activity, potentially enhancing anabolic sensitivity under conditions of metabolic stress [106].

In addition, polyphenols have been implicated in the regulation of autophagy, a cellular quality-control process essential for the removal of damaged proteins and organelles [107]. Properly regulated autophagy is critical for maintaining muscle integrity and metabolic function, particularly during aging [108]. By supporting balanced autophagic flux, polyphenols may contribute to improved muscle quality rather than indiscriminate increases in muscle mass [103].

Together, these findings suggest that polyphenols influence skeletal muscle health through coordinated regulation of protein synthesis, degradation, and cellular quality control mechanisms [103]. These effects provide a biological rationale for considering polyphenol-rich foods as complementary components of multimodal strategies aimed at preserving muscle function during aging [109]. Table 2 summarizes key polyphenols and polyphenol-rich food sources, their principal molecular targets, and reported effects on skeletal muscle, highlighting differences in the strength and scope of available evidence. Although much of the current evidence derives from mechanistic and early-phase human studies, the convergence of these pathways underscores their translational potential and supports further targeted clinical investigation in sarcopenia.

### 5.5. Polyphenol Metabolism and Bioavailability

Interpretation of the biological effects of dietary polyphenols requires consideration of their extensive metabolic transformation following ingestion [110]. A substantial proportion of consumed polyphenols does not reach target tissues in their native form but undergoes biotransformation mediated largely by the gut microbiota. Microbial metabolism generates lower-molecular-weight derivatives that often display enhanced bioavailability and distinct biological activities [111].

Following absorption, both parent compounds and microbiota-derived metabolites are further processed in enterocytes and the liver via conjugation reactions—including glucuronidation, sulfation, and methylation—thereby shaping their predominant circulating forms [112]. Emerging evidence suggests that these conjugated metabolites, rather than the original compounds, may represent key mediators of observed physiological effects [113].

Polyphenol bioavailability exhibits substantial interindividual variability, influenced by gut microbiome composition, dietary context, dose, and food matrix interactions. Rather than constituting a limitation alone, this variability reflects the context-dependent nature of polyphenol biology and may partly explain differences observed across human intervention studies. Accordingly, future clinical trials should incorporate standardized characterization of intake, metabolite profiling, and bioavailability assessment to enhance translational clarity [114].

## 6. Potential Complementary Mechanisms Between Polyphenol-Rich Foods and Resistance Training

In the context of this review, the interaction between polyphenol-rich dietary patterns and resistance training is interpreted as complementary biological processes identified primarily in mechanistic and experimental models rather than as established additive clinical effects. Current evidence derives largely from preclinical and exercise-based human studies; therefore, the proposed interactions are discussed as biologically plausible mechanisms with potential translational relevance. Polyphenol-rich diets and resistance training may converge on shared regulatory pathways governing skeletal muscle adaptation, recovery, and aging-related remodeling, providing a conceptual framework for understanding how nutritional and mechanical stimuli may jointly influence muscle physiology across the lifespan [115].

### 6.1. Modulation of Anabolic Resistance

Anabolic resistance, defined as the attenuated stimulation of muscle protein synthesis in response to anabolic stimuli such as amino acid intake and mechanical loading, is a central hallmark of age-related sarcopenia [116]. Chronic low-grade inflammation, impaired insulin sensitivity, and dysregulated redox homeostasis contribute to blunted activation of the mechanistic target of mTORC1 pathway in aging skeletal muscle [40].

Resistance training is a potent physiological stimulus for muscle protein synthesis; however, its anabolic efficacy may be diminished in older adults due to these age-associated constraints [117]. Accumulating evidence suggests that dietary polyphenols may indirectly contribute to enhanced anabolic responsiveness of skeletal muscle by attenuating inflammatory signaling and improving metabolic sensitivity [103,115,118]. Several polyphenolic compounds have been shown to downregulate pro-inflammatory mediators, including NF-κB, TNF-α, and IL-6, all of which are known to interfere with anabolic signaling cascades [91].

By mitigating inflammation-driven anabolic resistance and potentially improving amino acid sensing and leucine responsiveness, polyphenol-rich foods may enhance the muscle’s sensitivity to mechanical loading [119]. Importantly, these effects should be interpreted as modulatory rather than directly anabolic, underscoring the role of dietary polyphenols as facilitators of exercise-induced adaptations rather than standalone anabolic agents [120]. Current evidence supporting these interactions is primarily derived from mechanistic studies and a limited number of human investigations, and therefore, the clinical significance of these effects in sarcopenia remains to be confirmed.

### 6.2. Enhancement of Mitochondrial Adaptations and Metabolic Flexibility

Mitochondrial dysfunction and reduced metabolic flexibility are characteristic features of aging skeletal muscle and contribute to impaired contractile performance, delayed recovery, and increased fatigability [121]. Resistance training is known to induce mitochondrial adaptations, including increased mitochondrial content and enhanced oxidative capacity; however, these responses may be attenuated with advancing age [122].

Dietary polyphenols have been shown to influence mitochondrial biogenesis and function through the activation of key regulatory pathways, including AMP-activated protein kinase, sirtuin 1, and peroxisome proliferator-activated receptor gamma coactivator 1-alpha (PGC-1α) [123]. Rather than acting as direct antioxidants, many polyphenols function as mild metabolic stressors, eliciting adaptive cellular responses consistent with the concept of mitohormesis [124].

The combination of resistance training-induced mechanical stress and polyphenol-induced metabolic signaling may therefore act in a complementary manner to enhance mitochondrial adaptations. This interaction may improve energy efficiency and fatigue resistance in aging skeletal muscle, particularly in individuals with pre-existing mitochondrial impairment, for whom exercise alone may be insufficient to fully restore metabolic flexibility [125]. While these mechanisms are biologically plausible and supported by experimental evidence, human studies examining combined interventions remain limited and heterogeneous, precluding firm conclusions regarding clinical outcomes.

### 6.3. Regulation of Redox Balance and Adaptive Stress Responses

ROS play a dual role in skeletal muscle physiology, acting as damaging agents at excessive concentrations while serving as essential signaling molecules at physiological levels [126]. Exercise-induced ROS production is a critical driver of training adaptations; however, aging is associated with dysregulated redox homeostasis and increased susceptibility to oxidative damage [126].

A key consideration when combining dietary polyphenols with resistance training is the potential interference with exercise-induced redox signaling. Indeed, high-dose antioxidant supplementation has been shown to blunt adaptive responses to exercise, raising concerns regarding excessive antioxidant intake in physically active populations [127].

In contrast, polyphenol-rich whole foods may help modulate redox balance without abolishing ROS-mediated signaling [128]. Through the activation of endogenous antioxidant defense systems, including nuclear factor erythroid 2-related factor 2 (Nrf2)-dependent pathways, polyphenols may support adaptive stress responses while preserving the redox signals necessary for exercise-induced remodeling. This nuanced regulation of redox homeostasis may represent one potential mechanistic explanation for the complementary interaction between polyphenol intake and resistance training [94,128]. Importantly, the magnitude of these effects in real-world dietary contexts remains uncertain, as bioavailability, dose, and dietary patterns substantially influence physiological responses.

### 6.4. Effects on Muscle Quality and Neuromuscular Function

Beyond muscle mass, muscle quality—defined by strength per unit muscle mass, neuromuscular efficiency, and functional performance—is a critical determinant of mobility and independence in older adults. Resistance training effectively improves neuromuscular function; however, age-related impairments in motor unit recruitment, neuromuscular junction integrity, and excitation–contraction coupling may persist [129,130].

Emerging evidence suggests that polyphenols may exert beneficial effects on neuromuscular health through anti-inflammatory, vasoprotective, and neuroprotective mechanisms [103,131]. By attenuating neuroinflammation and supporting mitochondrial function within both motor neurons and muscle fibers, polyphenol-rich diets may complement resistance training-induced improvements in muscle quality and functional capacity [132]. These interactions provide a biologically plausible framework, particularly relevant to functional performance and recovery processes. Table 3 summarizes the key molecular and physiological pathways underlying these effects and is intended to inform future translational and clinical research rather than to serve as definitive clinical guidance.

## 7. Clinical Evidence in Humans

### 7.1. Resistance Training Combined with Polyphenol-Rich Foods

Studies conducted in young athletic populations primarily provide mechanistic insight and should not be directly generalized to sarcopenic older adults. This distinction is particularly relevant when interpreting post-exercise recovery studies, as the physiological processes governing acute recovery differ from the chronic mechanisms underlying sarcopenia. Accordingly, the evidence presented here is discussed within the context of exercise physiology and recovery, with implications for sarcopenia considered from a translational standpoint.

Over the past decade, human studies suggest that consumption of whole polyphenol-rich foods in combination with exercise may be associated with favorable effects on muscle function, post-exercise recovery, and selected aspects of training adaptation [133]. Evidence from randomized controlled trials indicates that green tea catechins, cocoa flavonoids, and various berry-derived polyphenols—particularly Montmorency tart cherry, blackcurrant, and blueberry—can attenuate markers of exercise-induced muscle damage, reduce delayed-onset muscle soreness (DOMS), and accelerate the recovery of muscle strength and functional performance, primarily within the first 24–72 h following exercise [134,135,136,137,138,139,140,141,142,143,144]. However, effect sizes are generally modest and not consistently observed across studies, and findings should be interpreted in light of differences in study design, dosage, and participant characteristics. These effects are commonly attributed to reductions in oxidative stress and inflammatory responses, as well as favorable modulation of redox homeostasis [143,145].

Several studies have reported that polyphenol-rich fruit juices and extracts, such as pomegranate, grape juice, açaí, and lychee, when combined with exercise, may enhance recovery following both endurance and resistance exercise, with performance benefits observed in some cases [103,146,147,148,149,150,151]. Beyond isolated foods or supplements, complex dietary patterns—most notably the Mediterranean diet—have also been associated with improved muscle function, lower frailty risk, and more favorable cardiometabolic profiles in older adults [11,13,152,153,154,155,156,157,158]. However, these observational findings reflect overall dietary patterns rather than isolated polyphenol effects and should be interpreted accordingly. These observations suggest that the physiological effects of polyphenols may be most pronounced when embedded within an overall high-quality dietary context [159,160,161].

More recent evidence indicates that polyphenol-rich, plant-based protein sources and snacks consumed alongside exercise may support not only muscle recovery but also the preservation of muscle mass and improvements in metabolic parameters. These effects may be mediated, at least in part, through modulation of the gut microbiota and its metabolic activity [134,135,136,162,163]. Nevertheless, substantial heterogeneity exists across studies with respect to polyphenol sources, doses, exercise modalities, intervention duration, and study populations, resulting in variable effect sizes and limiting direct comparisons across trials. Despite these limitations, the combination of exercise with food-based, polyphenol-rich dietary strategies appears to represent a biologically plausible approach that warrants further clinical investigation, particularly in populations at increased risk of functional decline. Detailed characteristics and outcomes of the relevant clinical studies are summarized in Table 4. Many of the studies summarized in Table 4 were conducted in healthy or physically active populations and should therefore be interpreted primarily as providing mechanistic or translational insight rather than direct clinical evidence in sarcopenic older adults, and should not be interpreted as evidence of efficacy in the treatment of sarcopenia. In particular, findings from recovery-focused protocols cannot be directly extrapolated to the pathophysiology of age-related muscle loss.

### 7.2. Supplement-Based Studies: Benefits and Limitations

Human randomized trials combining polyphenol supplementation with exercise show heterogeneous outcomes that are strongly dependent on polyphenol type and dose, intervention duration, the nature of the exercise stimulus, and the characteristics of the studied population [139,163,171]. Importantly, most available trials were conducted in healthy, physically active, or recreationally trained individuals; therefore, the findings primarily inform physiological and recovery responses to exercise rather than providing direct evidence for the prevention or treatment of sarcopenia.

During acute or short-term exercise challenges, several studies have reported performance- and recovery-related benefits. In endurance-trained athletes, Montmorency tart cherry supplementation improved race performance, attenuated the IL-6 response and muscle soreness, and enhanced antioxidant status [139]. Similarly, acute polyphenol-rich blends increased anaerobic peak power and reduced cardiovascular strain during high-intensity exercise [172]. Hesperidin supplementation improved nitric oxide bioavailability, endothelial function, and sprint or anaerobic performance, while VO_2_max generally remained unchanged [173]. These findings suggest that polyphenols may influence vascular, inflammatory, and redox-sensitive pathways relevant to short-term exercise responses and recovery.

In contrast, the effects of polyphenol supplementation during chronic exercise interventions are less consistent. Multiple studies indicate that exercise itself is the dominant adaptive stimulus and that supplementation does not consistently amplify it. In older adults, tart cherry- and resveratrol-based supplements did not enhance exercise- or protein-induced muscle protein synthesis [171], and in some settings, resveratrol attenuated exercise-induced mitochondrial and anti-inflammatory adaptations [163]. Conversely, other trials reported modest functional or mitochondrial benefits when resveratrol was combined with exercise, although these effects were not consistently accompanied by improvements in cardiometabolic outcomes [174,175].

Flavonoid-based interventions (e.g., quercetin glycosides) generally did not promote additional muscle hypertrophy, yet favorable neuromuscular and mechanical adaptations were observed, such as reduced muscle stiffness or improved motor unit activation [176,177]. Complex protein–polyphenol formulations enhanced early functional adaptations and myofibrillar protein synthesis but did not consistently translate into greater gains in muscle mass [178]. In other studies, polyphenol blends modulated exercise-induced oxidative and apoptotic signaling without impairing long-term strength adaptations [179,180]. Overall, polyphenol supplementation appears to confer the most consistent benefits in the context of acute exercise and recovery responses [139,172], whereas its influence on chronic training adaptations—particularly muscle hypertrophy and mitochondrial remodeling—remains limited and highly population dependent [163,171] (Table 5). In metabolically healthy individuals, antioxidant-oriented supplementation may attenuate redox-sensitive training adaptations, highlighting the importance of dose, timing, and context-specific application. Collectively, current clinical evidence supports a modulatory role of polyphenols in exercise-related physiological and short-term recovery processes. While definitive evidence for clinically meaningful effects on sarcopenia outcomes has yet to be established, the convergence of mechanistic and functional findings provides a rationale for further targeted investigation.

### 7.3. Target Populations and Personalized Approaches

The effectiveness of nutrition- and supplement-based interventions combined with exercise appears to be influenced by the biological and functional characteristics of the target population, underscoring the need for a precision nutrition framework for aging- and muscle-loss-related conditions. However, it should be emphasized that the current evidence base remains limited, and most findings should be interpreted as hypothesis-generating and exploratory rather than definitive guidance for clinical practice. Based on available human evidence, older adults living with sarcopenia, frailty, or functional limitations represent potentially relevant target populations, as suggested by clinical studies evaluating polyphenol-based interventions combined with exercise (Table 6). Nevertheless, the number of well-controlled trials specifically conducted in sarcopenic populations remains relatively small, and the heterogeneity of study designs and outcome measures limits firm conclusions.

In sarcopenic and obese sarcopenic individuals, intervention responses often differ from those observed in non-sarcopenic populations, highlighting the role of underlying metabolic and inflammatory status [182,183,184]. Across several studies, gains in muscle mass were modest, whereas improvements in muscle strength, neuromuscular function, or physical performance were more consistently observed [159,164,185,186]. These findings suggest that polyphenol-related interventions may influence functional and physiological parameters that are relevant to muscle health, although clear disease-modifying effects on sarcopenia have not yet been established.

In older but not necessarily sarcopenic populations, intervention effects were predominantly reflected in functional outcomes—such as gait speed, exercise tolerance, and quality of life—while changes in classical anabolic endpoints remained limited [165,171,174,175,187]. This pattern is consistent with the concept that, in later life, muscle quality and neuromuscular control may represent more sensitive and clinically meaningful targets than muscle mass alone. In frail older adults, response patterns appear even more heterogeneous; nevertheless, even modest functional improvements may be clinically meaningful for preserving independence and reducing the risk of disability [125,161]. Importantly, these functional endpoints may be more responsive to combined lifestyle interventions than structural muscle outcomes.

Emerging evidence further indicates that the gut microbiome may play a role in modulating individual responsiveness to exercise and nutritional interventions, potentially contributing to the distinction between responders and non-responders [162]. However, this area remains in an early stage of investigation and requires confirmation in larger longitudinal studies. Overall, the available evidence indicates that the effects of combined exercise and nutritional or supplement-based strategies are highly population-specific, emphasizing the importance of precise target population definition and the development of personalized approaches that integrate age, sex, functional status, dietary patterns, and microbiome-related factors. At present, these approaches should be regarded primarily as research directions rather than established clinical recommendations.

**Table 6:** Human intervention studies on polyphenol supplementation, alone or combined with exercise training, and their effects on muscle mass, strength, and functional outcomes in older and sarcopenic adults.

Author (Year)	Study Design	Population	Intervention—Training	Intervention—Polyphenol	Duration	Main Outcomes	Key Findings
Aubertin-Leheudre et al. (2007) [182]	Randomized, double-blind, placebo-controlled trial	Obese–sarcopenic postmenopausal women (50–70 y)	None	Soy isoflavones (70 mg/day: daidzein, genistein, glycitein)	6 months	Appendicular FFM, MMI (DXA)	Isoflavones increased appendicular and leg FFM and MMI vs. placebo; sarcopenia not fully reversed
Kim et al. (2013) [164]	Randomized, assessor-blinded, 4-arm RCT	Sarcopenic community-dwelling women ≥75 y	Multicomponent exercise (strength, balance, gait), 2×/week	Tea catechins (green tea), 540 mg/day	3 months	Muscle mass, gait speed, TUG, strength	Exercise + catechins improved leg muscle mass and walking speed more consistently than either intervention alone
Mafi et al. (2019) [185]	Randomized, double-blind, placebo-controlled, 4-arm trial	Sarcopenic older men (65–75 y)	Progressive resistance training, 3×/week	Epicatechin, 1 mg/kg/day	8 weeks	Strength, AppMMI, TUG, follistatin, myostatin	RT + epicatechin elicited the greatest gains in strength and anabolic signaling (↑ follistatin, ↓ myostatin)
Tokuda and Mori (2023) [186]	Open-label, pilot randomized controlled trial	Older adults with sarcopenia ≥ 65 y (AWGS 2019)	Elastic-band and body-weight resistance exercise, 2×/week	Tea catechins 540 mg/session + EAAs (3 g; leucine 1.2 g)	24 weeks	SMM, strength, gait speed, physical QOL	RE + EAA + catechins increased SMM, strength, gait speed and QOL vs. RE alone
Kwon et al. (2021) [159]	Pilot randomized, placebo-controlled trial	Older adults with sarcopenia ≥ 65 y	None	Marine oligomeric polyphenols (Ecklonia cava), ~72 mg/day	4 weeks	SMM, lean mass, balance	Polyphenols increased SMM, lean mass and balance without significant strength changes
Munguia et al. (2019) [165]	Double-blind, randomized, placebo-controlled trial	Older adults (55–90 y), pre-frail/frail	Daily walking (~30 min/day)	Cocoa flavonoids (~179 mg/day)	8–12 weeks	Mobility, SMI, inflammation, QoL	Cocoa flavonoids improved mobility, SMI and QoL and reduced oxidative stress and IL-6

Abbreviations: AppMMI, appendicular muscle mass index; AWGS, Asian Working Group for Sarcopenia; CSA, cross-sectional area; DXA, dual-energy X-ray absorptiometry; EAA, essential amino acids; FFM, fat-free mass; IL-6, interleukin-6; MMI, muscle mass index; QOL, quality of life; RE, resistance exercise; RCT, randomized controlled trial; RT, resistance training; SMI, skeletal muscle index; SMM, skeletal muscle mass; SPPB, Short Physical Performance Battery; TUG, Timed Up and Go test; VO_2_max, maximal oxygen uptake; ↑ indicates increase; ↓ indicates decrease.

## 8. Discussion

This review integrates mechanistic, translational, and human clinical evidence on the physiological and recovery-related effects of dietary polyphenols in the context of exercise, with particular emphasis on their relevance to muscle aging. Rather than positioning polyphenols as direct therapeutic agents for sarcopenia, we interpret them within the framework of exercise-activated processes—namely redox regulation, inflammatory modulation, mitochondrial adaptation, and neuromuscular recovery—and examine how these pathways intersect with established mechanisms of age-related muscle decline. To our knowledge, this review represents one of the first attempts to systematically align recovery-focused polyphenol physiology with the core pathophysiological domains of skeletal muscle aging, thereby proposing a unified, physiology-based translational framework. A substantial proportion of the available evidence derives from preclinical and early translational studies; clinical trials have been conducted across diverse populations, dosing regimens, and exercise protocols, limiting the formulation of definitive practice recommendations. In this context, the interaction between dietary polyphenols and exercise is interpreted as reflecting complementary biological processes rather than additive hypertrophic effects.

The chemical and biological diversity of polyphenols reflects real-world dietary exposure, as these compounds are typically consumed within whole-food matrices. Although flavonoids, stilbenes, and phenolic acids differ in pharmacokinetic properties and molecular targets, they converge on shared exercise-relevant signaling nodes—particularly redox-sensitive, inflammatory, and mitochondrial pathways. Accordingly, emphasis is placed on identifying convergent mechanisms rather than ranking individual compounds, thereby linking exercise-recovery physiology to core domains of sarcopenia pathophysiology.

Sarcopenia results from the interplay of anabolic resistance, chronic low-grade inflammation, oxidative stress, mitochondrial dysfunction, and neuromuscular impairment [188,189,190]. Resistance training remains the cornerstone of prevention and management; however, adaptive responses are often attenuated in older adults due to reduced molecular responsiveness [191]. This provides a conceptual, hypothesis-generating rationale for adjunctive strategies aimed at optimizing the cellular environment in which exercise adaptations occur [192].

Progressive RT improves muscle mass and functional performance [193]; however, inter-individual variability is substantial, and older adults often exhibit blunted muscle protein synthesis responses [194,195,196,197]. This suggests that age-related muscle decline reflects not only reduced mechanical loading but also a dysregulated adaptive milieu. Polyphenol-rich dietary patterns, including the Mediterranean diet, have been associated in observational studies with lower risk of frailty and improved functional status [198,199]. Polyphenols may exert pleiotropic effects through modulation of redox-sensitive and inflammatory signaling pathways [200], and when consumed within whole foods, their biological activity may be influenced by complex nutrient interactions [154,184,200,201].

The combination of RT and polyphenol-rich diets may contribute to physiological processes relevant to skeletal muscle adaptation through complementary mechanisms [132]. While RT provides the primary anabolic stimulus, polyphenols may help support recovery efficiency and adaptive responsiveness by modulating redox homeostasis and inflammatory signaling. Proposed mechanisms include activation of the AMPK–SIRT1–PGC-1α axis and restoration of redox balance [192]. Human studies indicate that these interactions may translate predominantly into functional benefits—such as improvements in muscle strength, gait performance, and endurance—while increases in muscle mass remain modest [32,125,134,135,136,137,138,139,140,141,142,144,147,148,149,150,151,159,160,161,162,163,164,165,166,167,168,169,170,171,172,173,174,175,176,177,178,179,180,181,182,185,186]. This supports the emerging paradigm that muscle quality and functional capacity represent clinically meaningful targets beyond hypertrophy alone. Systematic reviews further substantiate the biological plausibility of polyphenol-mediated recovery modulation [26,202]. In older clinical populations, structural adaptations appear limited, whereas functional improvements may be more pronounced [199].

From a clinical perspective, integrating polyphenol-rich dietary strategies with structured RT reinforces the systemic and metabolically mediated nature of sarcopenia [203]. A food-first approach emphasizing berries, green tea, extra-virgin olive oil, soy products, cocoa, legumes, and nuts may complement protein-centered nutritional strategies [204]. Although definitive disease-modifying evidence remains limited, aligning exercise-recovery physiology with aging-related muscle pathways provides a robust conceptual foundation for future hypothesis-driven clinical trials [205].

## 9. Limitations

Several limitations should be acknowledged. First, the number of controlled intervention studies conducted specifically in sarcopenic or frail older adults remains limited. Many trials are characterized by small sample sizes, short durations, and substantial heterogeneity in design and outcome measures. Therefore, current conclusions regarding clinical efficacy should be considered preliminary. Second, polyphenol exposure is inconsistently quantified, and isolated supplementation does not fully reflect the complexity, bioavailability, and potential complementary or interacting effects of whole-food-based dietary patterns. Third, outcome measures vary widely across studies, complicating direct comparison and synthesis. Finally, interindividual variability—potentially influenced by sex, metabolic health, microbiome composition, and comorbidities—has not been systematically addressed. Because of the substantial heterogeneity in study designs, populations, interventions, and endpoints, a formal quantitative meta-analysis was not feasible. The narrative design was intentionally chosen to integrate mechanistic, translational, and early clinical evidence within a single conceptual framework, and the findings should therefore be interpreted as hypothesis-generating rather than confirmatory.

## 10. Future Research Directions

Future research should prioritize adequately powered, long-term randomized controlled trials in well-defined sarcopenic and frail populations, employing clinically meaningful functional endpoints. Standardization of polyphenol characterization, dose quantification, and bioavailability assessment is essential. Trials should incorporate both mechanistic biomarkers (e.g., inflammation, redox status, mitochondrial function) and functional outcomes to clarify translational relevance.

Greater emphasis is needed on compound-specific effects, dose–response relationships, and food-based interventions rather than isolated supplementation. Integration of microbiome profiling and metabolic phenotyping may enable the identification of responder subgroups and support precision nutrition strategies.

The incorporation of digital health technologies may enhance adherence monitoring, functional assessment, and ecological validity in future trials. Finally, implementation research is needed to determine how combined exercise and dietary strategies can be feasibly integrated into geriatric care and preventive health frameworks.

## 11. Conclusions

Current mechanistic and early-phase clinical evidence suggests that combining resistance training with polyphenol-rich dietary strategies represents a biologically coherent approach that may help support exercise adaptation and functional performance during aging. Through coordinated modulation of redox homeostasis, inflammatory signaling, mitochondrial regulation, and neuromuscular processes, polyphenols may contribute to physiological recovery and adaptive efficiency, even when gains in muscle mass are modest. Although disease-modifying effects in sarcopenia remain to be established, the convergence of mechanistic and emerging clinical findings provides a translational, hypothesis-generating rationale for considering polyphenol-rich dietary patterns as part of multimodal lifestyle strategies centered on resistance training and adequate protein intake, rather than viewing them as standalone therapies. It highlights the need for rigorously designed clinical trials to clarify their clinical relevance.

## Figures and Tables

**Table 1:** Classification and dietary sources of polyphenols.

Polyphenol Class	Main Subclasses/Representative Compounds	Primary Dietary Sources (Whole Foods)	Notes on Bioavailability and Relevance to Skeletal Muscle
Flavonoids	Flavonols (quercetin, kaempferol); Flavanols (catechins, epicatechins); Flavones (luteolin); Flavanones (hesperidin); Isoflavones (genistein); Anthocyanins	Fruits (berries, apples, citrus), vegetables (onions, leafy greens), tea, cocoa, legumes	Most abundant dietary polyphenols; generally low bioavailability. However, biologically active metabolites may modulate inflammation, redox signaling, and muscle metabolism
Stilbenes	Resveratrol, piceatannol	Grapes, red wine, berries, peanuts	Present in relatively low dietary amounts; extensively studied for effects on mitochondrial function and metabolic regulation, mainly in preclinical models
Phenolic acids	Hydroxybenzoic acids (gallic acid); Hydroxycinnamic acids (caffeic acid, ferulic acid)	Coffee, whole grains, fruits, vegetables	Widely consumed; contribute to redox modulation and metabolic regulation; often present as conjugated forms
Lignans	Secoisolariciresinol, matairesinol	Flaxseed, whole grains, seeds, legumes	Converted by gut microbiota into bioactive metabolites; potential indirect effects on muscle metabolism
Other polyphenols	Tannins, ellagitannins	Nuts, pomegranates, berries, tea	Complex structures; biological effects largely mediated by microbial metabolites

Source: Own compilation.

**Table 2:** Polyphenols, molecular targets, and skeletal muscle outcomes.

Polyphenol/Polyphenol Class	Primary Molecular Targets and Pathways	Main Biological Effects on Skeletal Muscle	Level of Evidence
Flavonoids (e.g., quercetin, catechins)	Nuclear factor kappa-light-chain-enhancer of activated B cells; nuclear factor erythroid 2-related factor 2; adenosine monophosphate-activated protein kinase	Reduced inflammatory signaling; improved redox homeostasis; enhanced mitochondrial efficiency; potential improvement in fatigue resistance	Preclinical + limited human
Resveratrol (stilbene)	Sirtuin 1; adenosine monophosphate-activated protein kinase; peroxisome proliferator-activated receptor gamma coactivator 1 alpha	Increased mitochondrial biogenesis; improved metabolic flexibility; enhanced oxidative capacity; potential improvement in muscle endurance	Strong preclinical + heterogeneous human
Anthocyanins	Nuclear factor erythroid 2-related factor 2; mitogen-activated protein kinase pathways	Enhanced antioxidant defense via signaling; reduced exercise-induced oxidative stress; potential improvement in muscle recovery	Preclinical + emerging human
Phenolic acids (e.g., caffeic acid, ferulic acid)	Nuclear factor kappa-light-chain-enhancer of activated B cells; redox-sensitive signaling pathways	Modulation of inflammatory responses; support of metabolic homeostasis; indirect effects on muscle function	Mainly preclinical
Isoflavones	Estrogen receptor-mediated signaling; phosphatidylinositol 3-kinase–protein kinase B signaling	Modulation of muscle protein metabolism; potential preservation of muscle mass, particularly in postmenopausal populations	Human observational + limited trials
Polyphenol mixtures (whole foods)	Multi-target modulation of inflammatory, redox, and metabolic pathways	Potential improvements in muscle quality; support of stress resilience; possible complementary effects with exercise	Human observational + intervention

Source: Own compilation based on preclinical and human studies identified through the structured literature search described in Section 2.

**Table 3:** Proposed complementary mechanisms linking polyphenol-rich foods and resistance training in the context of skeletal muscle physiology, recovery, and aging.

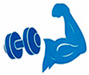 **Resistance training**	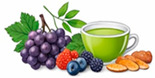 **Polyphenol-rich foods**
■ Mechanical loading ■ mTOR activation ■ Satellite cell activation ■ Exercise-induced ROS	■ Anti-inflammatory signaling (↓ NF-κB, TNF-α) ■ AMPK–SIRT1–PGC-1α activation ■ Redox balance (Nrf2 activation)
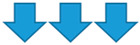	
**Proposed interacting mechanisms**	
■ Reduced anabolic resistance (indirect modulation) ■ Support of mitochondrial function ■ Preservation of adaptive redox signaling ■ Potential support of neuromuscular efficiency	
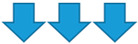	
**Potential physiological outcomes**	
Potential support of recovery and functional performance Improvement in muscle quality-related parameters Mechanistically supported relevance for muscle aging processes	

Source: Own compilation. Abbreviations: AMPK, adenosine monophosphate-activated protein kinase; mTOR, mechanistic target of rapamycin; NF-κB, nuclear factor kappa B; Nrf2, nuclear factor erythroid 2-related factor 2; PGC-1α, peroxisome proliferator-activated receptor gamma coactivator 1-alpha; ROS, reactive oxygen species; SIRT1, sirtuin 1; TNF-α, tumor necrosis factor alpha. ↓ indicates decrease.

**Table 4:** Clinical studies investigating the combined effects of polyphenols and exercise on skeletal muscle outcomes.

Author (Year)	Study Design	Population	Intervention—Training	Intervention—Polyphenol (Type/Source/Dose)	Duration	Main Outcomes	Key Findings
(A) Tea- and cocoa-derived polyphenols							
Jówko et al. (2011) [134]	RCT, double-blind, placebo-controlled	Untrained young men (*n* = 35)	Strength endurance training, 3×/week	Green tea extract, 640 mg polyphenols/day	4 weeks	Oxidative stress, antioxidant status, CK	↑ TAS and plasma polyphenols; ↓ lipid peroxidation; ↓ CK vs. placebo; ↔ SOD
Kuo et al. (2015) [135]	RCT, double-blind, placebo-controlled	Sedentary young men (*n* = 40)	Endurance training, 3×/week (75% VO_2_R, 20 min/session)	Green tea extract (catechins), 250 mg/day	4 weeks	Endurance performance, VO_2_max, oxidative stress, antioxidant status, CK	↑ VO_2_max and time to exhaustion (training groups); ↑ TAS (Ex + GTE); ↓ exercise-induced MDA and CK; ↔ training adaptations
Rahimi and Falahi (2017) [136]	RCT, double-blind, placebo-controlled, crossover	Obese men (*n* = 10)	Acute resistance exercise (75% 1RM, multiple exercises to exhaustion)	Green tea extract (≈400 mg catechins/day; EGCG-rich)	2 weeks (+ acute pre-RE dose)	Oxidative DNA damage (8-OHdG), lipid peroxidation (8-iso PGF_2_α)	↓ exercise-induced oxidative DNA damage (8-OHdG) vs. placebo; ↔ lipid peroxidation
Kim et al. (2013) [164]	RCT, assessor-blinded, parallel-group	Community-dwelling sarcopenic elderly women (≥75 y; *n* = 128 randomized)	Multicomponent exercise (strength, balance, gait), 2×/week	Tea catechins (catechin-fortified tea), 540 mg/day	3 months	Muscle mass, muscle strength, walking ability (gait speed, TUG)	↑ leg muscle mass and walking speed in exercise + catechin group; modest effects with exercise alone; ↔ muscle strength
Munguia et al. (2019) [165]	Double-blind, randomized, placebo-controlled clinical trial	Older men and women (55–70 y; follow-up: 65–90 y)	Daily walking recommendation (~30 min/day)	Cocoa flavonoids (natural cocoa), 179 mg flavonoids/day (epicatechin-rich)	12 weeks (follow-up: 8 weeks)	Oxidative stress, inflammation, mobility, muscle index, QoL	↓ lipid peroxidation and protein carbonyls; ↓ IL-6; ↑ skeletal muscle index, mobility (6MWT, TUG), and QoL; ↓ pre-frailty prevalence
da Silva et al. (2018) [144]	RCT, triple-blind, placebo-controlled	Untrained young men (*n* = 20)	Acute eccentric calf-raising exercise (DOMS induction)	Green tea extract (catechins), 500 mg/day	15 days	DOMS, muscle damage (CK, LDH), oxidative stress, antioxidant status	↓ CK at rest and post-exercise vs. placebo; ↔ DOMS, oxidative stress, and antioxidant markers
(B) Berry- and other fruit-derived plant sources							
Bell et al. (2016) [137]	RCT, double-blind, placebo-controlled	Semi-professional male soccer players (*n* = 16)	Prolonged intermittent sprint exercise (LISTADAPT)	Montmorency tart cherry concentrate, 30 mL twice/day	7 days	Muscle function, DOMS, inflammation, oxidative stress, CK	↑ recovery of muscle function; ↓ DOMS and IL-6; ↔ CK and lipid hydroperoxides
Howatson et al. (2010) [140]	RCT, double-blind, placebo-controlled	Recreational marathon runners, men and women (*n* = 20)	Marathon running (42.2 km)	Tart cherry juice blend, 2 × 240 mL/day (~600 mg phenolics/day)	8 days	Muscle function, DOMS, inflammation, oxidative stress, antioxidant status	↑ recovery of isometric strength; ↓ IL-6, CRP; ↑ total antioxidant status; ↓ TBARS; ↔ CK, LDH, DOMS
Quinlan and Hill (2020) [138]	Randomized, single-blind, placebo-controlled	Team sport athletes, men and women (*n* = 20)	Intermittent running (LIST)	Tart cherry juice concentrate, 30 mL twice/day	8 days	Muscle function, DOMS, CK, CRP	↑ faster recovery of CMJ, sprint, MVIC; ↓ soreness (trend); ↔ CK, CRP
Hooper et al. (2021) [142]	Randomized, placebo-controlled, crossover	Resistance-trained men (*n* = 13)	Acute resistance exercise (back squat, ~80% 1RM)	Tart cherry extract (NordicCherry^®^), 500 mg/day	7 days + acute bout	Oxidative stress, muscle damage, strength recovery	↓ protein carbonyls, CK, CK-MB; ↑ handgrip recovery; ↔ soreness, jump power
Hunt et al. (2021) [166]	RCT, double-blind, placebo-controlled	Non-resistance trained adults (*n* = 27)	Strenuous eccentric–concentric elbow exercise	NZ blackcurrant extract, 300 mg/day (105 mg anthocyanins)	12 days	Muscle function, soreness, CK, ROM	↑ MVC recovery; ↓ soreness and CK; ↔ ROM
Brandenburg and Giles (2019) [141]	Randomized, double-blind, crossover	Recreational runners (*n* = 14)	8 km running time trial	Blueberry powder (anthocyanin-rich)	4 days	Performance, lactate, neuromuscular function	↔ performance; ↓ post-exercise lactate; attenuated RSI decline
Carvalho-Peixoto et al. (2015) [151]	Randomized, single-blind, crossover	Elite male athletes (*n* = 14)	Maximal treadmill running (90% VO_2_max)	Açai beverage, 300 mL (27.6 mg anthocyanins)	Acute + 3-day loading	Muscle stress, oxidative stress, RPE, TTE	↑ time-to-exhaustion; ↓ RPE; ↓ CK, LDH, MDA; ↑ GPx
Ostojic et al. (2008) [167]	Randomized, placebo-controlled trial	College athletes (*n* = 20)	Habitual training	Coffeeberry extract, 800 mg/day	4 weeks	Antioxidant capacity, performance, recovery	↑ total antioxidant capacity; ↑ HR recovery; ↓ lactate during recovery; ↔ VO_2_max
Toscano et al. (2015) [149]	Randomized, controlled trial	Recreational runners (*n* = 28)	Habitual running + lab endurance tests	Purple grape juice, 10 mL/kg/day	28 days	Time-to-exhaustion, oxidative stress, inflammation	↑ time-to-exhaustion; ↑ antioxidant capacity; ↓ α-1-acid glycoprotein; ↔ VO_2_max
Torregrosa-García et al. (2019) [150]	Double-blind, randomized, placebo-controlled, crossover	Endurance-trained male cyclists (*n* = 26)	SWEET + IETE + eccentric exercise	Pomegranate extract, 225 mg punicalagins/day	15 days/arm	Performance, VT2, recovery, CK, CRP	↑ time-to-exhaustion and VT2; ↔ VO_2_max; trend ↓ CK, CRP
Nishizawa et al. (2011) [147]	Randomized, double-blind, placebo-controlled trial	Young male long-distance runners (*n* = 20)	High-intensity endurance training	Flavanol-rich lychee extract, 100 mg/day	2 months	Inflammation, oxidative stress, muscle damage	↓ IL-6 early; ↑ TGF-β1 post-training; ↓ resting HR; ↔ CK
Kang et al. (2012) [148]	Double-blind, randomized, placebo-controlled (3-arm)	Recreationally active men (*n* = 59)	Aerobic exercise + treadmill test	Oligomerized lychee extract, 200 mg/day	30 days	Endurance, threshold, VO_2_max	↑ submaximal TTE and anaerobic threshold; ↔ VO_2_max (OLFE); ↓ VO_2_max with vit C+E
(C) Complex nutritional interventions							
Flensted-Jensen et al. (2025) [125]	Randomized, double-blind, placebo-controlled	Healthy older adults, men and women, 55–70 y (*n* = 41)	Supervised resistance training (3×/week) + minimal HIIT (1×/week)	Red- and blackcurrant-derived polyphenols (~700 mg/day)	30-day loading + 12 weeks	Body composition, muscle strength, VO_2_max, metabolic, and inflammatory markers	RT+HIIT ↑ lean mass, strength and VO_2_max; ↓ submaximal HR, lactate and cortisol; attenuated exercise-induced IL-10, IFN-γ and TNF-α; polyphenols alone ↓ cholesterol without additive training effects
d’Unienville et al. (2025) [168]	Randomized, single-blind, controlled trial	Recreationally trained male cyclists (*n* = 90 analyzed)	Periodized endurance cycling (light → heavy → taper)	Polyphenol-rich snack (almonds 75 g + dried grapes 25 g + cranberries 25 g/day)	5 weeks	Endurance performance, NO bioavailability, oxidative stress, muscle damage, recovery	↑ nitric oxide bioavailability; ↓ RER and ↑ fat oxidation during submaximal exercise; ↑ perceived energy and recovery; ↔ 5 min TT performance and VO_2_peak vs. control
Kawamura et al. (2021) [160]	Randomized controlled trial	Healthy untrained young men (*n* = 26)	Resistance training, 2×/week (whole body, 10 RM)	Astaxanthin-, β-carotene-, and resveratrol-rich foods (salmon flakes, vegetable juice, lingonberry jam)	10 weeks	Muscle strength, body composition, metabolic rate, oxidative stress, fatigue	RT ↑ muscle mass in both groups; combined polyphenol-rich foods ↑ MVC, ↑ resting oxygen consumption, ↓ subjective fatigue; trend toward ↓ exercise-induced protein carbonylation
Carrera-Quintanar et al. (2015) [169]	Single-blind, randomized, parallel-group RCT	Young trained male rowers, 20–22 y	Supervised eccentric resistance training, 3×/week	Lippia citriodora extract (PLX^®^, 1.2 g/day), vitamin C+E-enriched almond beverage, or combination	3 weeks	Redox status, antioxidant enzymes, oxidative stress markers	Eccentric training ↑ oxidative stress; PLX^®^ (± almond beverage) attenuated lipid/protein oxidation, preserved SOD and GRD activity, ↓ myoglobin; no clear performance effects
Nieman et al. (2013) [170]	Randomized, double-blind, placebo-controlled, parallel-group	Endurance-trained runners, men and women, 19–45 y (*n* = 31 completed)	Intensified endurance running (2.5 h/day × 3 days, ~70% VO_2_max)	Polyphenol–soy protein complex (blueberry + green tea), 40 g/day (~2136 mg GAE/day)	17 days (14 pre + 3 exercise)	Inflammation, oxidative stress, antioxidant capacity, metabolomics	↔ exercise-induced inflammation and oxidative stress vs. placebo; ↑ gut-derived phenolic metabolites; ↑ fat oxidation and ketone bodies during recovery; no clear performance benefit
Chang et al. (2023) [162]	Quasi-experimental, open-label, single-arm pre–post	Community-dwelling elderly with low muscle mass, ≥65 y (*n* = 46)	None (habitual activity)	Fermented black soybean polyphenol-rich protein (BSKP), 2 packs/day (~16 g protein/day; isoflavone-rich)	10 weeks	Muscle mass, lipid profile, antioxidant enzymes, gut microbiota	↑ appendicular muscle mass and ASMI; ↓ LDL; ↑ catalase, GPx and SOD; gut microbiota remodeling with ↑ SCFAs; ↔ muscle strength and gait speed
Clayton-Chubb et al. (2024) [161]	Cross-sectional secondary analysis (RCT + cohort)	Community-dwelling older adults ≥ 70 y, Australia (*n* ≈ 12,400)	None (observational)	Mediterranean Diet adherence (ASPREE-MDS) and UPF intake (ASPREE-UPF), FFQ-derived	~3 years post-baseline	Frailty index, cardiometabolic conditions	Higher MedDiet adherence associated with ↓ pre-frailty/frailty, ↓ hypertension and CKD; higher UPF intake associated with ↑ frailty; weak inverse MedDiet–UPF relationship

Abbreviations: 1RM, one-repetition maximum; 6MWT, 6-min walk test; 8-OHdG, 8-hydroxy-2′-deoxyguanosine; ASMI, appendicular skeletal muscle mass index; BSKP, black soybean polyphenol-rich protein; CK, creatine kinase; CKD, chronic kidney disease; CMJ, countermovement jump; CRP, C-reactive protein; DOMS, delayed-onset muscle soreness; EGCG, epigallocatechin gallate; Ex, exercise; FFQ, food frequency questionnaire; GAE, gallic acid equivalents; GPx, glutathione peroxidase; GRD, glutathione reductase; GTE, green tea extract; HIIT, high-intensity interval training; HR, heart rate; IFN-γ, interferon gamma; IL-6, interleukin 6; IL-10, interleukin 10; LDH, lactate dehydrogenase; LDL, low-density lipoprotein cholesterol; LIST, Loughborough Intermittent Shuttle Test; MDA, malondialdehyde; MVC, maximal voluntary contraction; NO, nitric oxide; PGF2α, prostaglandin F2 alpha; PLX^®^, Lippia citriodora extract; QoL, quality of life; RCT, randomized controlled trial; RE, resistance exercise; RER, respiratory exchange ratio; ROM, range of motion; RPE, rating of perceived exertion; SCFAs, short-chain fatty acids; SOD, superoxide dismutase; TAS, total antioxidant status; TNF-α, tumor necrosis factor alpha; TT, time trial; TTE, time to exhaustion; TUG, Timed Up and Go test; UPF, ultra-processed food; VO_2_max, maximal oxygen uptake; VO_2_peak, peak oxygen uptake; VO_2_R, oxygen uptake reserve; VT2, second ventilatory threshold. Symbols: ↑ indicates increase; ↓ indicates decrease; ↔ indicates no significant change.

**Table 5:** Exercise–polyphenol interaction studies: effects on performance, muscle adaptations, and oxidative stress markers.

Author (Year)	Study Design	Population	Intervention—Training	Intervention—Polyphenol	Duration	Main Outcomes	Key Findings
Pavis et al. (2022) [178]	RCT, double-blind, placebo-controlled	Healthy recreationally active adults (*n* = 29)	Unilateral resistance training (~3×/week)	Protein–polyphenol beverage (pomegranate + tart cherry extracts; ~1.1 g/day)	~10 weeks	MyoPS, muscle function, hypertrophy	↑ MyoPS and early functional gains; ↑ type II fiber CSA; no additional whole-muscle hypertrophy vs. placebo
Alway et al. (2017) [175]	RCT, double-blind, placebo-controlled	Healthy older adults (65–80 y; *n* = 30)	Combined aerobic + resistance training	Resveratrol, 500 mg/day	12 weeks	Muscle strength, fatigue resistance, mitochondrial density	Resveratrol + exercise ↑ mitochondrial density, fatigue resistance, strength, power, fiber CSA and myonuclei vs. exercise alone; no added cardiometabolic benefit
Beyer et al. (2017) [179]	RCT, double-blind, placebo-controlled	Untrained young men (18–31 y; *n* = 40)	Progressive full-body resistance training, 3×/week	Tea-derived polyphenol blend, 2000 mg/day	4-week loading + 6 weeks RT	Antioxidant capacity, muscle damage, strength	↑ total antioxidant capacity; no attenuation of training-induced strength gains
Imperatrice et al. (2022) [173]	RCTs, double-blind, placebo-controlled	Recreationally trained adults (*n* ≈ 15–40)	Endurance- and sprint-based exercise tests	Hesperidin, 217–500 mg (acute) or 360–500 mg/day	Acute to 8 weeks	Endothelial function, oxidative stress, performance	↑ NO bioavailability and endothelial function; ↓ oxidative stress and inflammation; ↑ anaerobic and sprint performance; ↔ VO_2_max
Townsend et al. (2018) [180]	Randomized, placebo-controlled	Untrained young men (*n* = 38)	Acute high-volume resistance exercise	Tea-derived polyphenol blend, 2 g/day	28 days + acute bout	Intramuscular apoptotic signaling	Polyphenols modulated early apoptotic signaling post-exercise without preventing muscle damage responses
Levers et al. (2016) [139]	RCT, double-blind, placebo-controlled	Endurance-trained runners and triathletes (*n* = 27)	Half-marathon (21.1 km)	Montmorency tart cherry powder, 480 mg/day (~66 mg anthocyanins)	10 days	Performance, muscle damage, inflammation	↑ race performance; ↓ IL-6 and muscle soreness; ↑ antioxidant status; ↔ CK, TBARS
Harper et al. (2021) [174]	Pilot RCT, triple-masked	Older adults ≥65 y with functional limitations (*n* = 60)	Walking + resistance training, 2×/week	Resveratrol 500 or 1000 mg/day	12 weeks	Physical function, strength, mitochondrial markers	Exercise + resveratrol safe and feasible; modest functional improvements, greatest at 1000 mg; no clear additive anti-inflammatory effect
Otsuka et al. (2022) [176]	RCT, double-blind, parallel-group	Physically inactive adults 50–74 y (*n* = 48)	Low-intensity resistance training (40% 1RM), 3×/week	Quercetin glycosides 200 or 500 mg/day	24 weeks	Muscle CSA, stiffness, lean mass	No added hypertrophy vs. training; ↓ VL muscle stiffness independent of CSA; safe
Nishikawa et al. (2025) [177]	RCT, double-blind	Healthy older adults 65–82 y (*n* = 26)	Isometric knee extension, 3×/week	Quercetin glycosides 200 mg/day	6 weeks	Strength, motor unit behavior	↑ strength gains and high-threshold motor unit firing vs. training alone; ↔ muscle mass
Scholten et al. (2015) [181]	RCT, double-blind, parallel-group	Physically active men 25–45 y (*n* = 35)	Habitual training maintained	Quercetin 1000 mg/day ± vitamin D_3_	8 weeks	VO_2_max, strength, redox markers	No improvement in fitness or strength; minor antioxidant changes without functional benefit
Cases et al. (2017) [172]	RCT, double-blind, crossover	Recreationally active young men (*n* = 15)	Acute Wingate cycling (4 × 30 s)	Polyphenol-rich extract (PerfLoad^®^), 1000 mg acute	Acute	Anaerobic power, redox stress	↑ peak and mean power (~3–5%); ↓ cardiovascular strain; ↔ fatigue index
Jackman et al. (2018) [171]	RCT, double-blind	Healthy older men 60–75 y (*n* = 16)	Unilateral RT + protein (10 g/day)	Montmorency cherry concentrate (~540 mg anthocyanins/day)	3 weeks	Myofibrillar protein synthesis	No enhancement of resting or exercise-stimulated MPS; exercise + protein robustly ↑ MPS
Olesen et al. (2014) [163]	RCT, double-blind (exercise × supplement)	Physically inactive older men 60–72 y (*n* = 43)	Endurance + circuit training	Resveratrol 250 mg/day	8 weeks	Endurance, mitochondrial enzymes, inflammation	Exercise markedly improved metabolic and inflammatory markers; resveratrol alone ineffective and blunted some training adaptations

Abbreviations: RCT, randomized controlled trial; y, years; n, sample size; mg, milligrams; day, per day; RT, resistance training; 1RM, one-repetition maximum; CSA, cross-sectional area; NO, nitric oxide; VO_2_max, maximal oxygen uptake; IL-6, interleukin-6; CK, creatine kinase; TBARS, thiobarbituric acid–reactive substances; VL, vastus lateralis; MPS, myofibrillar protein synthesis; ↑, increase/improvement; ↓, decrease; ↔, no change.

## Data Availability

No new data were created or analyzed in this study.

## References

[1] Hawley J.A., Lundby C., Cotter J.D., Burke L.M. (2018). Maximizing cellular adaptation to endurance exercise in skeletal muscle. Cell Metab..

[2] Bessa A.L., Oliveira V.N., Agostini G.G., Oliveira R.J., Oliveira A.C., White G.E., Wells G.D., Teixeira D.N., Espindola F.S. (2016). Exercise intensity and recovery: Biomarkers of injury, inflammation, and oxidative stress. J. Strength Cond. Res..

[3] El Assar M., Álvarez-Bustos A., Sosa P., Angulo J., Rodríguez-Mañas L. (2022). Effect of physical activity/exercise on oxidative stress and inflammation in muscle and vascular aging. Int. J. Mol. Sci..

[4] Egan B., Sharples A.P. (2023). Molecular responses to acute exercise and their relevance for adaptations in skeletal muscle to exercise training. Physiol. Rev..

[5] Martin A., Prior R., Shukitt-Hale B., Cao G., Joseph J.A. (2000). Effect of fruits, vegetables, or vitamin E–rich diet on vitamins E and C distribution in peripheral and brain tissues: Implications for brain function. J. Gerontol. Ser. A Biol. Sci. Med. Sci..

[6] Houston D.K. (2020). The Role of Diet on Life and Health Span—Lessons Learned over the Past 75 Years.

[7] Vidaček N.Š., Nanić L., Ravlić S., Sopta M., Gerić M., Gajski G., Garaj-Vrhovac V., Rubelj I. (2018). Telomeres, nutrition, and longevity: Can we really navigate our aging?. J. Gerontol. Ser. A.

[8] Nikolov J., Spira D., Aleksandrova K., Otten L., Meyer A., Demuth I., Steinhagen-Thiessen E., Eckardt R., Norman K. (2016). Adherence to a Mediterranean-style diet and appendicular lean mass in community-dwelling older people: Results from the Berlin Aging Study II. J. Gerontol. Ser. A Biomed. Sci. Med. Sci..

[9] Vince F.-P., Zoltán U., Mónika F. (2024). Táplálkozási stratégiák az egészséges öregedésért: Krónikus, életkorral összefüggő betegségek. Sci. J. Hung. Assoc. Gerontol. Geriatr..

[10] Madarász B., Fazekas-Pongor V., Szarvas Z., Fekete M., Varga J.T., Tarantini S., Csiszar A., Lionetti V., Tabák A.G., Ungvari Z. (2024). Survival and longevity of European rulers: Geographical influences and exploring potential factors, including the Mediterranean diet—A historical analysis from 1354 to the twentieth century. GeroScience.

[11] Fekete M., Csípő T., Fazekas-Pongor V., Bálint M., Csizmadia Z., Tarantini S., Varga J.T. (2023). The Possible Role of Food and Diet in the Quality of Life in Patients with COPD-A State-of-the-Art Review. Nutrients.

[12] Ungvari Z., Fekete M., Varga P., Lehoczki A., Fekete J.T., Ungvari A., Győrffy B. (2025). Overweight and obesity significantly increase colorectal cancer risk: A meta-analysis of 66 studies revealing a 25–57% elevation in risk. GeroScience.

[13] Fekete M., Varga P., Ungvari Z., Fekete J.T., Buda A., Szappanos Á., Lehoczki A., Mózes N., Grosso G., Godos J. (2025). The role of the Mediterranean diet in reducing the risk of cognitive impairement, dementia, and Alzheimer’s disease: A meta-analysis. Geroscience.

[14] Scalbert A., Johnson I.T., Saltmarsh M. (2005). Polyphenols: Antioxidants and beyond. Am. J. Clin. Nutr..

[15] Manful C.F., Fordjour E., Subramaniam D., Sey A.A., Abbey L., Thomas R. (2025). Antioxidants and reactive oxygen species: Shaping human health and disease outcomes. Int. J. Mol. Sci..

[16] Fekete M., Lehoczki A., Kryczyk-Poprawa A., Zábó V., Varga J.T., Bálint M., Fazekas-Pongor V., Csípő T., Rząsa-Duran E., Varga P. (2025). Functional Foods in Modern Nutrition Science: Mechanisms, Evidence, and Public Health Implications. Nutrients.

[17] Mileo A.M., Miccadei S. (2016). Polyphenols as modulator of oxidative stress in cancer disease: New therapeutic strategies. Oxidative Med. Cell. Longev..

[18] Murakami A. (2024). Impact of hormesis to deepen our understanding of the mechanisms underlying the bioactivities of polyphenols. Curr. Opin. Biotechnol..

[19] Davinelli S., De Stefani D., De Vivo I., Scapagnini G. (2020). Polyphenols as caloric restriction mimetics regulating mitochondrial biogenesis and mitophagy. Trends Endocrinol. Metab..

[20] Serrano J.C., Cassanye A., Martín-Gari M., Granado-Serrano A.B., Portero-Otín M. (2016). Effect of dietary bioactive compounds on mitochondrial and metabolic flexibility. Diseases.

[21] Tippairote T., Hoonkaew P., Suksawang A., Tippairote P. (2025). From adaptation to exhaustion: Defining exposure-related malnutrition as a bioenergetic phenotype of aging. Biogerontology.

[22] Meng Q., Su C.-H. (2025). Antioxidant Defense and Redox Signaling in Elite Soccer Players: Insights into Muscle Function, Recovery, and Training Adaptations. Antioxidants.

[23] Trouwborst I., Verreijen A., Memelink R., Massanet P., Boirie Y., Weijs P., Tieland M. (2018). Exercise and nutrition strategies to counteract sarcopenic obesity. Nutrients.

[24] Phillips S.M. (2015). Nutritional supplements in support of resistance exercise to counter age-related sarcopenia. Adv. Nutr..

[25] Kan N.-W., Lee M.-C., Tung Y.-T., Chiu C.-C., Huang C.-C., Huang W.-C. (2018). The synergistic effects of resveratrol combined with resistant training on exercise performance and physiological adaption. Nutrients.

[26] Martinez-Negrin G., Acton J.P., Cocksedge S.P., Bailey S.J., Clifford T. (2022). The effect of dietary (poly) phenols on exercise-induced physiological adaptations: A systematic review and meta-analysis of human intervention trials. Crit. Rev. Food Sci. Nutr..

[27] Ciupei D., Colişar A., Leopold L., Stănilă A., Diaconeasa Z.M. (2024). Polyphenols: From classification to therapeutic potential and bioavailability. Foods.

[28] Fiore M., Tonchev A.B., Pancheva R.Z., Yamashima T., Venditti S., Ferraguti G., Terracina S. (2025). Increasing life expectancy with plant polyphenols: Lessons from the Mediterranean and Japanese diets. Molecules.

[29] Peterson M.D., Sen A., Gordon P.M. (2011). Influence of resistance exercise on lean body mass in aging adults: A meta-analysis. Med. Sci. Sports Exerc..

[30] Jang H., Song J., Kim J., Lee H., Lee H., Park H.-y., Shin H., Kwon Y.-e., Kim Y., Yim J. (2025). The Present and Future of Sarcopenia Diagnosis and Exercise Interventions: A Narrative Review. Appl. Sci..

[31] Qiu H., Zheng W., Zhou X., Liu Q., Zhao X. (2025). Training modalities for elder sarcopenic obesity: A systematic review and network meta-analysis. Front. Nutr..

[32] Ungvari Z., Fekete M., Varga P., Munkácsy G., Fekete J.T., Lehoczki A., Buda A., Kiss C., Ungvari A., Győrffy B. (2025). Exercise and survival benefit in cancer patients: Evidence from a comprehensive meta-analysis. GeroScience.

[33] Ryall C., Denham J. (2025). A systematic review and meta-analysis highlights a link between aerobic fitness and telomere maintenance. J. Gerontol. Ser. A Biol. Sci. Med. Sci..

[34] Sindi S., Solomon A., Kåreholt I., Hovatta I., Antikainen R., Hänninen T., Levälahti E., Laatikainen T., Lehtisalo J., Lindström J. (2021). Telomere length change in a multidomain lifestyle intervention to prevent cognitive decline: A randomized clinical trial. J. Gerontol. Ser. A.

[35] Carey J.R., Liedo P., Müller H.-G., Wang J.-L., Chiou J.-M. (1998). Relationship of age patterns of fecundity to mortality, longevity, and lifetime reproduction in a large cohort of Mediterranean fruit fly females. J. Gerontol. Ser. A Biol. Sci. Med. Sci..

[36] Noren Hooten N., Mode N.A., Valipour S., Zonderman A.B., Evans M.K. (2025). The interface of geroscience with longitudinal health disparities research: A 20-year retrospective of the Healthy Aging in Neighborhoods of Diversity across the Life Span study. J. Gerontol. Ser. A Biol. Sci. Med. Sci..

[37] Breen L., Phillips S.M. (2011). Skeletal muscle protein metabolism in the elderly: Interventions to counteract the ‘anabolic resistance’ of ageing. Nutr. Metab..

[38] Brook M., Wilkinson D., Phillips B., Perez-Schindler J., Philp A., Smith K., Atherton P. (2016). Skeletal muscle homeostasis and plasticity in youth and ageing: Impact of nutrition and exercise. Acta Physiol..

[39] Tu S., Hao X., Xu S., Jin X., Liao W., Xia H., Wang S., Sun G. (2025). Sarcopenia: Current insights into molecular mechanisms, diagnostics, and emerging interventional approaches. Int. J. Mol. Sci..

[40] Pérez-Castillo Í.M., Rueda R., Pereira S.L., Bouzamondo H., López-Chicharro J., Segura-Ortiz F., Atherton P.J. (2025). Age-Related Anabolic Resistance: Nutritional and Exercise Strategies, and Potential Relevance to Life-Long Exercisers. Nutrients.

[41] Aragon A.A., Tipton K.D., Schoenfeld B.J. (2023). Age-related muscle anabolic resistance: Inevitable or preventable?. Nutr. Rev..

[42] Ryall J.G., Schertzer J.D., Lynch G.S. (2008). Cellular and molecular mechanisms underlying age-related skeletal muscle wasting and weakness. Biogerontology.

[43] Sirago G., Picca A., Calvani R., Coelho-Júnior H.J., Marzetti E. (2022). Mammalian target of rapamycin (mTOR) signaling at the crossroad of muscle fiber fate in sarcopenia. Int. J. Mol. Sci..

[44] Salminen A., Kaarniranta K., Kauppinen A. (2021). Insulin/IGF-1 signaling promotes immunosuppression via the STAT3 pathway: Impact on the aging process and age-related diseases. Inflamm. Res..

[45] Henrique Mazucanti C., Victor Cabral-Costa J., Rodrigues Vasconcelos A., Zukas Andreotti D., Scavone C., Mitiko Kawamoto E. (2015). Longevity pathways (mTOR, SIRT, Insulin/IGF-1) as key modulatory targets on aging and neurodegeneration. Curr. Top. Med. Chem..

[46] Cuthbertson D., Smith K., Babraj J., Leese G., Waddell T., Atherton P., Wackerhage H., Taylor P.M., Rennie M.J. (2005). Anabolic signaling deficits underlie amino acid resistance of wasting, aging muscle. FASEB J..

[47] Drummond M.J., Dreyer H.C., Pennings B., Fry C.S., Dhanani S., Dillon E.L., Sheffield-Moore M., Volpi E., Rasmussen B.B. (2008). Skeletal muscle protein anabolic response to resistance exercise and essential amino acids is delayed with aging. J. Appl. Physiol..

[48] Damanti S., Azzolino D., Roncaglione C., Arosio B., Rossi P., Cesari M. (2019). Efficacy of nutritional interventions as stand-alone or synergistic treatments with exercise for the management of sarcopenia. Nutrients.

[49] Martínez-Arnau F.M., Fonfría-Vivas R., Buigues C., Castillo Y., Molina P., Hoogland A.J., van Doesburg F., Pruimboom L., Fernández-Garrido J., Cauli O. (2020). Effects of leucine administration in sarcopenia: A randomized and placebo-controlled clinical trial. Nutrients.

[50] Dalle S., Rossmeislova L., Koppo K. (2017). The role of inflammation in age-related sarcopenia. Front. Physiol..

[51] Sharma B., Dabur R. (2020). Role of pro-inflammatory cytokines in regulation of skeletal muscle metabolism: A systematic review. Curr. Med. Chem..

[52] Ma W., Xu T., Wang Y., Wu C., Wang L., Yang X., Sun H. (2018). The role of inflammatory factors in skeletal muscle injury. Biotarget.

[53] Vella L., Caldow M.K., Larsen A.E., Tassoni D., Della Gatta P.A., Gran P., Russell A.P., Cameron-Smith D. (2012). Resistance exercise increases NF-κB activity in human skeletal muscle. Am. J. Physiol.-Regul. Integr. Comp. Physiol..

[54] Salminen A., Kaarniranta K. (2009). NF-κB signaling in the aging process. J. Clin. Immunol..

[55] Williamson J., Davison G. (2020). Targeted antioxidants in exercise-induced mitochondrial oxidative stress: Emphasis on DNA damage. Antioxidants.

[56] Bouviere J., Fortunato R.S., Dupuy C., Werneck-de-Castro J.P., Carvalho D.P., Louzada R.A. (2021). Exercise-stimulated ROS sensitive signaling pathways in skeletal muscle. Antioxidants.

[57] Arcaro A., Lepore A., Cetrangolo G.P., Paventi G., Ames P.R.J., Gentile F. (2025). A reassessment of sarcopenia from a redox perspective as a basis for preventive and therapeutic interventions. Int. J. Mol. Sci..

[58] Ferri E., Marzetti E., Calvani R., Picca A., Cesari M., Arosio B. (2020). Role of age-related mitochondrial dysfunction in sarcopenia. Int. J. Mol. Sci..

[59] Kim Y., Triolo M., Hood D.A. (2017). Impact of aging and exercise on mitochondrial quality control in skeletal muscle. Oxidative Med. Cell. Longev..

[60] Goodpaster B.H., Sparks L.M. (2017). Metabolic flexibility in health and disease. Cell Metab..

[61] Shoemaker M.E., Gillen Z.M., Fukuda D.H., Cramer J.T. (2023). Metabolic flexibility and inflexibility: Pathology underlying metabolism dysfunction. J. Clin. Med..

[62] Venditti P., Di Meo S. (2020). The role of reactive oxygen species in the life cycle of the mitochondrion. Int. J. Mol. Sci..

[63] Gan Z., Fu T., Kelly D.P., Vega R.B. (2018). Skeletal muscle mitochondrial remodeling in exercise and diseases. Cell Res..

[64] Bellanti F., Buglio A.L., Vendemiale G. (2020). Oxidative stress and sarcopenia. Aging.

[65] Beckwée D., Delaere A., Aelbrecht S., Baert V., Beaudart C., Bruyere O., de Saint-Hubert M., Bautmans I. (2019). Exercise interventions for the prevention and treatment of sarcopenia. A systematic umbrella review. J. Nutr. Health Aging.

[66] Talar K., Hernández-Belmonte A., Vetrovsky T., Steffl M., Kałamacka E., Courel-Ibáñez J. (2021). Benefits of resistance training in early and late stages of frailty and sarcopenia: A systematic review and meta-analysis of randomized controlled studies. J. Clin. Med..

[67] Hart P.D., Buck D.J. (2019). The effect of resistance training on health-related quality of life in older adults: Systematic review and meta-analysis. Health Promot. Perspect..

[68] Borde R., Hortobágyi T., Granacher U. (2015). Dose–response relationships of resistance training in healthy old adults: A systematic review and meta-analysis. Sports Med..

[69] Carroll T.J., Riek S., Carson R.G. (2001). Neural adaptations to resistance training: Implications for movement control. Sports Med..

[70] Attwaters M., Hughes S.M. (2022). Cellular and molecular pathways controlling muscle size in response to exercise. FEBS J..

[71] Gonzalez A.M., Hoffman J.R., Stout J.R., Fukuda D.H., Willoughby D.S. (2016). Intramuscular anabolic signaling and endocrine response following resistance exercise: Implications for muscle hypertrophy. Sports Med..

[72] Cartee G.D. (1994). Aging skeletal muscle: Response to exercise. Exerc. Sport Sci. Rev..

[73] Groennebaek T., Vissing K. (2017). Impact of resistance training on skeletal muscle mitochondrial biogenesis, content, and function. Front. Physiol..

[74] Porter C., Reidy P.T., Bhattarai N., Sidossis L.S., Rasmussen B.B. (2015). Resistance exercise training alters mitochondrial function in human skeletal muscle. Med. Sci. Sports Exerc..

[75] Zhao Y.-C., Wu Y.-Y. (2023). Resistance training improves hypertrophic and mitochondrial adaptation in skeletal muscle. Int. J. Sports Med..

[76] Grgic J., Garofolini A., Orazem J., Sabol F., Schoenfeld B.J., Pedisic Z. (2020). Effects of Resistance Training on Muscle Size and Strength in Very Elderly Adults: A Systematic Review and Meta-Analysis of Randomized Controlled Trials. Sports Med..

[77] Pickering C., Kiely J. (2019). Do non-responders to exercise exist—And if so, what should we do about them?. Sports Med..

[78] Rivera-Torres S., Fahey T.D., Rivera M.A. (2019). Adherence to exercise programs in older adults: Informative report. Gerontol. Geriatr. Med..

[79] Van Roie E., Bautmans I., Coudyzer W., Boen F., Delecluse C. (2015). Low-and high-resistance exercise: Long-term adherence and motivation among older adults. Gerontology.

[80] Kim D., Morikawa S., Miyawaki M., Nakagawa T., Ogawa S., Kase Y. (2025). Sarcopenia prevention in older adults: Effectiveness and limitations of non-pharmacological interventions. Osteoporos. Sarcopenia.

[81] Rathod N.B., Elabed N., Punia S., Ozogul F., Kim S.-K., Rocha J.M. (2023). Recent developments in polyphenol applications on human health: A review with current knowledge. Plants.

[82] Fekete M., Jarecsny T., Lehoczki A., Major D., Fazekas-Pongor V., Csípő T., Lipécz Á., Szappanos Á., Pázmándi E.M., Varga P. (2025). Mediterranean Diet, Polyphenols, and Neuroprotection: Mechanistic Insights into Resveratrol and Oleuropein. Nutrients.

[83] Garg S.K., Shukla A., Choudhury S. (2019). Polyphenols and flavonoids. Nutraceuticals in Veterinary Medicine.

[84] Brglez Mojzer E., Knez Hrnčič M., Škerget M., Knez Ž., Bren U. (2016). Polyphenols: Extraction methods, antioxidative action, bioavailability and anticarcinogenic effects. Molecules.

[85] Panche A.N., Diwan A.D., Chandra S.R. (2016). Flavonoids: An overview. J. Nutr. Sci..

[86] Cory H., Passarelli S., Szeto J., Tamez M., Mattei J. (2018). The role of polyphenols in human health and food systems: A mini-review. Front. Nutr..

[87] Martin K.R., Appel C.L. (2009). Polyphenols as dietary supplements: A double-edged sword. Nutr. Diet. Suppl..

[88] Aatif M. (2023). Current understanding of polyphenols to enhance bioavailability for better therapies. Biomedicines.

[89] Bolaños-Cardet J., Pepió-Tárrega B., Saiz-Poseu J., López-Moral A., Ullah F., Yuste V.J., Ruiz-Molina D., Suárez-García S. (2025). The Redox Properties of Polyphenols and Their Role in ROS Generation for Biomedical Applications. Angew. Chem..

[90] Singh A., Yau Y.F., Leung K.S., El-Nezami H., Lee J.C.-Y. (2020). Interaction of polyphenols as antioxidant and anti-inflammatory compounds in brain–liver–gut axis. Antioxidants.

[91] Yahfoufi N., Alsadi N., Jambi M., Matar C. (2018). The immunomodulatory and anti-inflammatory role of polyphenols. Nutrients.

[92] Centonze M., Aloisio Caruso E., De Nunzio V., Cofano M., Saponara I., Pinto G., Notarnicola M. (2025). The Antiaging Potential of Dietary Plant-Based Polyphenols: A Review on Their Role in Cellular Senescence Modulation. Nutrients.

[93] Zhou Y., Jiang Z., Lu H., Xu Z., Tong R., Shi J., Jia G. (2019). Recent advances of natural polyphenols activators for Keap1-Nrf2 signaling pathway. Chem. Biodivers..

[94] Scapagnini G., Sonya V., Nader A.G., Calogero C., Zella D., Fabio G. (2011). Modulation of Nrf2/ARE pathway by food polyphenols: A nutritional neuroprotective strategy for cognitive and neurodegenerative disorders. Mol. Neurobiol..

[95] Rao M.J., Zheng B. (2025). The role of polyphenols in abiotic stress tolerance and their antioxidant properties to scavenge reactive oxygen species and free radicals. Antioxidants.

[96] Rudrapal M., Khairnar S.J., Khan J., Dukhyil A.B., Ansari M.A., Alomary M.N., Alshabrmi F.M., Palai S., Deb P.K., Devi R. (2022). Dietary polyphenols and their role in oxidative stress-induced human diseases: Insights into protective effects, antioxidant potentials and mechanism (s) of action. Front. Pharmacol..

[97] Mthembu S.X., Dludla P.V., Ziqubu K., Nyambuya T.M., Kappo A.P., Madoroba E., Nyawo T.A., Nkambule B.B., Silvestri S., Muller C.J. (2021). The potential role of polyphenols in modulating mitochondrial bioenergetics within the skeletal muscle: A systematic review of preclinical models. Molecules.

[98] Wood dos Santos T., Cristina Pereira Q., Teixeira L., Gambero A., Villena J.A., Lima Ribeiro M. (2018). Effects of polyphenols on thermogenesis and mitochondrial biogenesis. Int. J. Mol. Sci..

[99] Stevens J.F., Revel J.S., Maier C.S. (2018). Mitochondria-centric review of polyphenol bioactivity in cancer models. Antioxid. Redox Signal..

[100] Stromsnes K., Lagzdina R., Olaso-Gonzalez G., Gimeno-Mallench L., Gambini J. (2021). Pharmacological properties of polyphenols: Bioavailability, mechanisms of action, and biological effects in in vitro studies, animal models, and humans. Biomedicines.

[101] Stefania D.S., Clodoveo M., Cariello M., D’Amato G., Franchini C., Faienza M., Corbo F. (2021). Polyphenols and obesity prevention: Critical insights on molecular regulation, bioavailability and dose in preclinical and clinical settings. Crit. Rev. Food Sci. Nutr..

[102] Xiang J., Du M., Wang H. (2023). Dietary plant extracts in improving skeletal muscle development and metabolic function. Food Rev. Int..

[103] Nikawa T., Ulla A., Sakakibara I. (2021). Polyphenols and their effects on muscle atrophy and muscle health. Molecules.

[104] Zanchi N.E., Lancha A.H. (2008). Mechanical stimuli of skeletal muscle: Implications on mTOR/p70s6k and protein synthesis. Eur. J. Appl. Physiol..

[105] Wu Q., Lv Q., Liu X.a., Ye X., Cao L., Wang M., Li J., Yang Y., Li L., Wang S. (2023). Natural compounds from botanical drugs targeting mTOR signaling pathway as promising therapeutics for atherosclerosis: A review. Front. Pharmacol..

[106] Cao Y., Han S., Lu H., Luo Y., Guo T., Wu Q., Luo F. (2022). Targeting mTOR signaling by dietary polyphenols in obesity prevention. Nutrients.

[107] Hu M., Liu W., Cao F., Jin S. (2025). Polyphenols and exercise in autophagy regulation: Potential benefits for cancer management and healthspan. Front. Nutr..

[108] Brimson J.M., Prasanth M.I., Malar D.S., Thitilertdecha P., Kabra A., Tencomnao T., Prasansuklab A. (2021). Plant polyphenols for aging health: Implication from their autophagy modulating properties in age-associated diseases. Pharmaceuticals.

[109] Numa I.A.N., Sancho R.A.S., Wolf K.E., da Silva Miranda C.T.C., Soares S.D., de Souza Lima A., Pastore G.M. (2025). Polyphenols, aging, and health: What can we expect from the food industry in the technology era?. Front. Med..

[110] Lu C., Zhang J., Zhao X., Zi Y., Xiao X. (2025). Biotransformation of Phenolic Acids in Foods: Pathways, Key Enzymes, and Technological Applications. Foods.

[111] Williamson G. (2025). Bioavailability of Food Polyphenols: Current State of Knowledge. Annu. Rev. Food Sci. Technol..

[112] Rudrapal M., de Oliveira A.M., Singh R.P. (2025). Dietary polyphenols maintain human health through modulation of gut microbiota. Front. Pharmacol..

[113] Mahdi L., Graziani A., Baffy G., Mitten E.K., Portincasa P., Khalil M. (2025). Unlocking polyphenol efficacy: The role of gut microbiota in modulating bioavailability and health effects. Nutrients.

[114] Bié J., Sepodes B., Fernandes P.C., Ribeiro M.H. (2023). Polyphenols in health and disease: Gut microbiota, bioaccessibility, and bioavailability. Compounds.

[115] Rezaei M.J. (2025). Synergistic effects of polyphenols and exercise on obesity: Targeting metabolism, muscle function, and adipose tissue remodeling. Front. Nutr..

[116] Tezze C., Sandri M., Tessari P. (2023). Anabolic resistance in the pathogenesis of sarcopenia in the elderly: Role of nutrition and exercise in young and old people. Nutrients.

[117] Hunter G.R., McCarthy J.P., Bamman M.M. (2004). Effects of resistance training on older adults. Sports Med..

[118] Capozzi A., Saucier C., Bisbal C., Lambert K. (2022). Grape polyphenols in the treatment of human skeletal muscle damage due to inflammation and oxidative stress during obesity and aging: Early outcomes and promises. Molecules.

[119] Rickards L., Lynn A., Harrop D., Barker M.E., Russell M., Ranchordas M.K. (2021). Effect of polyphenol-rich foods, juices, and concentrates on recovery from exercise induced muscle damage: A systematic review and meta-analysis. Nutrients.

[120] Malaguti M., Angeloni C., Hrelia S. (2013). Polyphenols in exercise performance and prevention of exercise-induced muscle damage. Oxidative Med. Cell. Longev..

[121] Grevendonk L., Connell N.J., McCrum C., Fealy C.E., Bilet L., Bruls Y.M., Mevenkamp J., Schrauwen-Hinderling V.B., Jörgensen J.A., Moonen-Kornips E. (2021). Impact of aging and exercise on skeletal muscle mitochondrial capacity, energy metabolism, and physical function. Nat. Commun..

[122] Parry H.A., Roberts M.D., Kavazis A.N. (2020). Human skeletal muscle mitochondrial adaptations following resistance exercise training. Int. J. Sports Med..

[123] Maksimović T., Gădău C., Antal G., Čoban M., Eșanu O., Atyim E., Mioc A., Șoica C. (2025). Polyphenol-Based Therapeutic Strategies for Mitochondrial Dysfunction in Aging. Biomolecules.

[124] Chodari L., Dilsiz Aytemir M., Vahedi P., Alipour M., Vahed S.Z., Khatibi S.M.H., Ahmadian E., Ardalan M., Eftekhari A. (2021). Targeting mitochondrial biogenesis with polyphenol compounds. Oxidative Med. Cell. Longev..

[125] Flensted-Jensen M., Weinreich C.M., Kleis-Olsen A.-S., Hansen F., Skyggelund N.S., Pii J.R., Whitlock R., Abrahamsen M.-L.B., Petersen T.I., Karlsen A. (2025). Effects of resistance-based training and polyphenol supplementation on physical function, metabolism, and inflammation in aging individuals. GeroScience.

[126] Powers S.K., Radak Z., Ji L.L., Jackson M. (2024). Reactive oxygen species promote endurance exercise-induced adaptations in skeletal muscles. J. Sport Health Sci..

[127] Canals-Garzón C., Guisado-Barrilao R., Martínez-García D., Chirosa-Ríos I.J., Jerez-Mayorga D., Guisado-Requena I.M. (2022). Effect of antioxidant supplementation on markers of oxidative stress and muscle damage after strength exercise: A systematic review. Int. J. Environ. Res. Public Health.

[128] Tkaczenko H., Kurhaluk N. (2025). Antioxidant-rich functional foods and exercise: Unlocking metabolic health through Nrf2 and related pathways. Int. J. Mol. Sci..

[129] Kendall H., Kipp L.E., Mettler J.A. (2025). Resistance Training Preserves Physical Function in Older Community-Dwelling Adults. Transl. J. Am. Coll. Sports Med..

[130] Reid K.F., Fielding R.A. (2012). Skeletal muscle power: A critical determinant of physical functioning in older adults. Exerc. Sport Sci. Rev..

[131] Petrella C., Di Certo M.G., Gabanella F., Barbato C., Ceci F.M., Greco A., Ralli M., Polimeni A., Angeloni A., Severini C. (2021). Mediterranean diet, brain and muscle: Olive polyphenols and resveratrol protection in neurodegenerative and neuromuscular disorders. Curr. Med. Chem..

[132] Zhang X., Zhong Y., Rajabi S. (2025). Polyphenols and post-exercise muscle damage: A comprehensive review of literature. Eur. J. Med. Res..

[133] Andreo-López M.C., Contreras-Bolívar V., Muñoz-Torres M., García-Fontana B., García-Fontana C. (2023). Influence of the Mediterranean diet on healthy aging. Int. J. Mol. Sci..

[134] Jówko E., Sacharuk J., Balasińska B., Ostaszewski P., Charmas M., Charmas R. (2011). Green tea extract supplementation gives protection against exercise-induced oxidative damage in healthy men. Nutr. Res..

[135] Kuo Y.-C., Lin J.-C., Bernard J.R., Liao Y.-H. (2015). Green tea extract supplementation does not hamper endurance-training adaptation but improves antioxidant capacity in sedentary men. Appl. Physiol. Nutr. Metab..

[136] Rahimi R., Falahi Z. (2017). Effect of green tea extract on exercise-induced oxidative stress in obese men: A randomized, double-blind, placebo-controlled, crossover study. Asian J. Sports Med..

[137] Bell P.G., Stevenson E., Davison G.W., Howatson G. (2016). The effects of montmorency tart cherry concentrate supplementation on recovery following prolonged, intermittent exercise. Nutrients.

[138] Quinlan R., Hill J.A. (2020). The efficacy of tart cherry juice in aiding recovery after intermittent exercise. Int. J. Sports Physiol. Perform..

[139] Levers K., Dalton R., Galvan E., O’Connor A., Goodenough C., Simbo S., Mertens-Talcott S.U., Rasmussen C., Greenwood M., Riechman S. (2016). Effects of powdered Montmorency tart cherry supplementation on acute endurance exercise performance in aerobically trained individuals. J. Int. Soc. Sports Nutr..

[140] Howatson G., McHugh M.P., Hill J., Brouner J., Jewell A., Van Someren K.A., Shave R., Howatson S. (2010). Influence of tart cherry juice on indices of recovery following marathon running. Scand. J. Med. Sci. Sports.

[141] Brandenburg J.P., Giles L.V. (2019). Four Days of Blueberry Powder Supplementation Lowers the Blood Lactate Response to Running But Has No Effect on Time-Trial Performance. Int. J. Sport Nutr. Exerc. Metab..

[142] Hooper D.R., Orange T., Gruber M.T., Darakjian A.A., Conway K.L., Hausenblas H.A. (2021). Broad Spectrum Polyphenol Supplementation from Tart Cherry Extract on Markers of Recovery from Intense Resistance Exercise. J. Int. Soc. Sports Nutr..

[143] Dehghani E., Beba M., Danandeh K., Memari A., Ershadmanesh M.J., Rasoulian P., Danandeh A., Djafarian K. (2025). The effect of tart cherry juice (TCJ) supplementation on exercise-induced muscle damage (EIMD) in an athletic population. Ann. Med. Surg..

[144] Da Silva W., Machado Á.S., Souza M.A., Mello-Carpes P.B., Carpes F.P. (2018). Effect of green tea extract supplementation on exercise-induced delayed onset muscle soreness and muscular damage. Physiol. Behav..

[145] Ruszkowska J., Drygas W., Kwaśniewska M. (2024). The Influence of Berry-Derived Polyphenol Supplementation on Exercise-Induced Oxidative Stress and Cardiovascular Health in Physically Active Individuals. Antioxidants.

[146] Liu S., Zhang L., Li S. (2023). Advances in nutritional supplementation for sarcopenia management. Front. Nutr..

[147] Nishizawa M., Hara T., Miura T., Fujita S., Yoshigai E., Ue H., Hayashi Y., Kwon A.H., Okumura T., Isaka T. (2011). Supplementation with a flavanol-rich lychee fruit extract influences the inflammatory status of young athletes. Phytother. Res..

[148] Kang S.W., Hahn S., Kim J.-K., Yang S.-M., Park B.-J., Lee S.C. (2012). Oligomerized lychee fruit extract (OLFE) and a mixture of vitamin C and vitamin E for endurance capacity in a double blind randomized controlled trial. J. Clin. Biochem. Nutr..

[149] Toscano L.T., Tavares R.L., Toscano L.T., Silva C.S.O.d., Almeida A.E.M.d., Biasoto A.C.T., Gonçalves M.d.C.R., Silva A.S. (2015). Potential ergogenic activity of grape juice in runners. Appl. Physiol. Nutr. Metab..

[150] Torregrosa-García A., Ávila-Gandía V., Luque-Rubia A.J., Abellán-Ruiz M.S., Querol-Calderón M., López-Román F.J. (2019). Pomegranate extract improves maximal performance of trained cyclists after an exhausting endurance trial: A randomised controlled trial. Nutrients.

[151] Carvalho-Peixoto J., Moura M.R.L., Cunha F.A., Lollo P.C.B., Monteiro W.D., Carvalho L.M.J.d., Farinatti P.d.T.V. (2015). Consumption of açai (*Euterpe oleracea* Mart.) functional beverage reduces muscle stress and improves effort tolerance in elite athletes: A randomized controlled intervention study. Appl. Physiol. Nutr. Metab..

[152] Fekete M., Lehoczki A., Major D., Fazekas-Pongor V., Csípő T., Tarantini S., Csizmadia Z., Varga J.T. (2024). Exploring the influence of gut–brain axis modulation on cognitive health: A comprehensive review of prebiotics, probiotics, and symbiotics. Nutrients.

[153] Fekete M., Szarvas Z., Fazekas-Pongor V., Feher A., Csipo T., Forrai J., Dosa N., Peterfi A., Lehoczki A., Tarantini S. (2022). Nutrition strategies promoting healthy aging: From improvement of cardiovascular and brain health to prevention of age-associated diseases. Nutrients.

[154] Ungvari Z., Fekete M., Fekete J.T., Grosso G., Ungvari A., Győrffy B. (2025). Adherence to the Mediterranean diet and its protective effects against colorectal cancer: A meta-analysis of 26 studies with 2,217,404 participants. Geroscience.

[155] Lehoczki A., Csípő T., Lipécz Á., Major D., Fazekas-Pongor V., Csík B., Mózes N., Fehér Á., Dósa N., Árva D. (2025). Western diet and cognitive decline: A Hungarian perspective—Implications for the design of the semmelweis study. Nutrients.

[156] Corina A., Abrudan M.B., Nikolic D., Cătoi A.F., Chianetta R., Castellino G., Citarrella R., Stoian A.P., Pérez-Martínez P., Rizzo M. (2019). Effects of aging and diet on cardioprotection and cardiometabolic risk markers. Curr. Pharm. Des..

[157] McEvoy C.T., McClure C.D. (2024). Nutrition Resilience for Healthy Ageing.

[158] Dreher M.L. (2018). Dietary Patterns and Whole Plant Foods in Aging and Disease.

[159] Kwon I.-S., Park D.-S., Shin H.-C., Seok M.-G., Oh J.-K. (2021). Effects of marine oligomeric polyphenols on body composition and physical ability of elderly individuals with sarcopenia: A pilot study. Phys. Act. Nutr..

[160] Kawamura A., Aoi W., Abe R., Kobayashi Y., Kuwahata M., Higashi A. (2021). Astaxanthin-, β-carotene-, and resveratrol-rich foods support resistance training-induced adaptation. Antioxidants.

[161] Clayton-Chubb D., Vaughan N.V., George E.S., Chan A.T., Roberts S.K., Ryan J., Phyo A.Z.Z., McNeil J.J., Beilin L.J., Tran C. (2024). Mediterranean Diet and Ultra-Processed Food Intake in Older Australian Adults—Associations with Frailty and Cardiometabolic Conditions. Nutrients.

[162] Chang S.-S., Chen L.-H., Huang K.-C., Huang S.-W., Chang C.-C., Liao K.-W., Hu E.-C., Chen Y.-P., Chen Y.-W., Hsu P.-C. (2023). Plant-based polyphenol rich protein supplementation attenuated skeletal muscle loss and lowered the LDL level via gut microbiota remodeling in Taiwan’s community-dwelling elderly. Food Funct..

[163] Olesen J., Gliemann L., Biensø R., Schmidt J., Hellsten Y., Pilegaard H. (2014). Exercise training, but not resveratrol, improves metabolic and inflammatory status in skeletal muscle of aged men. J. Physiol..

[164] Kim H., Suzuki T., Saito K., Yoshida H., Kojima N., Kim M., Sudo M., Yamashiro Y., Tokimitsu I. (2013). Effects of exercise and tea catechins on muscle mass, strength and walking ability in community-dwelling elderly Japanese sarcopenic women: A randomized controlled trial. Geriatr. Gerontol. Int..

[165] Munguia L., Rubio-Gayosso I., Ramirez-Sanchez I., Ortiz A., Hidalgo I., Gonzalez C., Meaney E., Villarreal F., Najera N., Ceballos G. (2019). High flavonoid cocoa supplement ameliorates plasma oxidative stress and inflammation levels while improving mobility and quality of life in older subjects: A double-blind randomized clinical trial. J. Gerontol. Ser. A.

[166] Hunt J.E., Coelho M.O., Buxton S., Butcher R., Foran D., Rowland D., Gurton W., Macrae H., Jones L., Gapper K.S. (2021). Consumption of New Zealand blackcurrant extract improves recovery from exercise-induced muscle damage in non-resistance trained men and women: A double-blind randomised trial. Nutrients.

[167] Ostojic S.M., Stojanovic M.D., Djordjevic B., Jourkesh M., Vasiljevic N. (2008). The effects of a 4-week coffeeberry supplementation on antioxidant status, endurance, and anaerobic performance in college athletes. Res. Sports Med..

[168] d’Unienville N.M.A., Coates A.M., Hill A.M., Nelson M.J., Croft K., Yandell C., Buckley J.D. (2025). Polyphenol-Rich Snack Consumption during Endurance Exercise Training Improves Nitric Oxide Bioavailability but does not Improve Exercise Performance in Male Cyclists: A Randomised Controlled Trial. Curr. Dev. Nutr..

[169] Carrera-Quintanar L., Funes L., Vicente-Salar N., Blasco-Lafarga C., Pons A., Micol V., Roche E. (2015). Effect of polyphenol supplements on redox status of blood cells: A randomized controlled exercise training trial. Eur. J. Nutr..

[170] Nieman D.C., Gillitt N.D., Knab A.M., Shanely R.A., Pappan K.L., Jin F., Lila M.A. (2013). Influence of a polyphenol-enriched protein powder on exercise-induced inflammation and oxidative stress in athletes: A randomized trial using a metabolomics approach. PLoS ONE.

[171] Jackman S.R., Brook M.S., Pulsford R.M., Cockcroft E.J., Campbell M.I., Rankin D., Atherton P., Smith K., Bowtell J.L. (2018). Tart cherry concentrate does not enhance muscle protein synthesis response to exercise and protein in healthy older men. Exp. Gerontol..

[172] Cases J., Romain C., Marín-Pagán C., Chung L.H., Rubio-Pérez J.M., Laurent C., Gaillet S., Prost-Camus E., Prost M., Alcaraz P.E. (2017). Supplementation with a Polyphenol-Rich Extract, PerfLoad(^®^), Improves Physical Performance during High-Intensity Exercise: A Randomized, Double Blind, Crossover Trial. Nutrients.

[173] Imperatrice M., Cuijpers I., Troost F.J., Sthijns M.M. (2022). Hesperidin functions as an ergogenic aid by increasing endothelial function and decreasing exercise-induced oxidative stress and inflammation, thereby contributing to improved exercise performance. Nutrients.

[174] Harper S.A., Bassler J.R., Peramsetty S., Yang Y., Roberts L.M., Drummer D., Mankowski R.T., Leeuwenburgh C., Ricart K., Patel R.P. (2021). Resveratrol and exercise combined to treat functional limitations in late life: A pilot randomized controlled trial. Exp. Gerontol..

[175] Alway S.E., McCrory J.L., Kearcher K., Vickers A., Frear B., Gilleland D.L., Bonner D.E., Thomas J.M., Donley D.A., Lively M.W. (2017). Resveratrol enhances exercise-induced cellular and functional adaptations of skeletal muscle in older men and women. J. Gerontol. Ser. A Biomed. Sci. Med. Sci..

[176] Otsuka Y., Miyamoto N., Nagai A., Izumo T., Nakai M., Fukuda M., Arimitsu T., Yamada Y., Hashimoto T. (2022). Effects of quercetin glycoside supplementation combined with low-intensity resistance training on muscle quantity and stiffness: A randomized, controlled trial. Front. Nutr..

[177] Nishikawa T., Takeda R., Ueda S., Igawa K., Hirono T., Okudaira M., Mita Y., Ohya T., Watanabe K. (2025). Quercetin ingestion alters motor unit behavior and enhances improvement in muscle strength following resistance training in older adults: A randomized, double-blind, controlled trial. Eur. J. Nutr..

[178] Pavis G.F., Jameson T.S., Blackwell J.R., Fulford J., Abdelrahman D.R., Murton A.J., Alamdari N., Mikus C.R., Wall B.T., Stephens F.B. (2022). Daily protein-polyphenol ingestion increases daily myofibrillar protein synthesis rates and promotes early muscle functional gains during resistance training. Am. J. Physiol.-Endocrinol. Metab..

[179] Beyer K.S., Stout J.R., Fukuda D.H., Jajtner A.R., Townsend J.R., Church D.D., Wang R., Riffe J.J., Muddle T.W., Herrlinger K.A. (2017). Impact of polyphenol supplementation on acute and chronic response to resistance training. J. Strength Cond. Res..

[180] Townsend J.R., Stout J.R., Jajtner A.R., Church D.D., Beyer K.S., Riffe J.J., Muddle T.W.D., Herrlinger K.L., Fukuda D.H., Hoffman J.R. (2018). Polyphenol supplementation alters intramuscular apoptotic signaling following acute resistance exercise. Physiol. Rep..

[181] Scholten S.D., Sergeev I.N., Song Q., Birger C.B. (2015). Effects of vitamin D and quercetin, alone and in combination, on cardiorespiratory fitness and muscle function in physically active male adults. Open Access J. Sports Med..

[182] Aubertin-Leheudre M., Lord C., Khalil A., Dionne I. (2007). Six months of isoflavone supplement increases fat-free mass in obese–sarcopenic postmenopausal women: A randomized double-blind controlled trial. Eur. J. Clin. Nutr..

[183] Jarecsny T., Egri C.A., Kosik R., Schwab R., Mechtler L., Szollosi G.J., Schandl L., Tomasics G., Gyuricsko I., Pazmandi E.M. (2026). Economic burden of stroke attributable to excess body mass in Hungary: A population-attributable fraction analysis. BMC Public Health.

[184] Cao X., Peng H., Hu Z., Xu C., Ning M., Zhou M., Mi Y., Yu P., Fazekas-Pongor V., Major D. (2025). Exploring the global impact of obesity and diet on dementia burden: The role of national policies and sex differences. Geroscience.

[185] Mafi F., Biglari S., Ghardashi Afousi A., Gaeini A.A. (2019). Improvement in Skeletal Muscle Strength and Plasma Levels of Follistatin and Myostatin Induced by an 8-Week Resistance Training and Epicatechin Supplementation in Sarcopenic Older Adults. J. Aging Phys. Act..

[186] Tokuda Y., Mori H. (2023). Essential amino acid and tea catechin supplementation after resistance exercise improves skeletal muscle mass in older adults with sarcopenia: An open-label, pilot, randomized controlled trial. J. Am. Nutr. Assoc..

[187] Mukli P., Muranyi M., Lipecz Á., Szarvas Z., Csípő T., Ungvari A., Fekete M., Fazekas-Pongor V., Peterfi A., Fehér Á. (2025). Age-related and dual task-induced gait alterations and asymmetry: Optimizing the Semmelweis Study gait assessment protocol. Geroscience.

[188] Cruz-Jentoft A.J., Bahat G., Bauer J., Boirie Y., Bruyère O., Cederholm T., Cooper C., Landi F., Rolland Y., Sayer A.A. (2019). Sarcopenia: Revised European consensus on definition and diagnosis. Age Ageing.

[189] Major D., Dósa N., Balázs P., Fekete M., Pártos K., Árva D., Mészáros Á., Terebessy A., Tabák Á.G., Fazekas-Pongor V. (2026). Global trends in the incidence and prevalence of Alzheimer’s disease. Advances in Translational Research. Adv. Transl. Res..

[190] Gulej R., Nagy D., Kristof R., Csiszar A., Patai R. (2026). Circulating factors as modifiable therapeutic targets in brain and cerebrovascular aging: Insights from heterochronic parabiosis. Adv. Transl. Res..

[191] Larsson L., Ramamurthy B. (2000). Aging-related changes in skeletal muscle: Mechanisms and interventions. Drugs Aging.

[192] Fraga C.G., Croft K.D., Kennedy D.O., Tomás-Barberán F.A. (2019). The effects of polyphenols and other bioactives on human health. Food Funct..

[193] Cadore E.L., Izquierdo M. (2013). How to simultaneously optimize muscle strength, power, functional capacity, and cardiovascular gains in the elderly: An update. Age.

[194] Kumar V., Selby A., Rankin D., Patel R., Atherton P., Hildebrandt W., Williams J., Smith K., Seynnes O., Hiscock N. (2009). Age-related differences in the dose–response relationship of muscle protein synthesis to resistance exercise in young and old men. J. Physiol..

[195] Degens H. (2010). The role of systemic inflammation in age-related muscle weakness and wasting. Scand. J. Med. Sci. Sports.

[196] Győrffy B., Szabo C., Ungvari Z. (2025). Welcome to Advances in Translational Research: Expanding the horizons of translational pharmacology. Adv. Transl. Res..

[197] Sorond F., Jea A., Hegyi P., Yabluchanskiy A. (2026). Guidelines for meta-analyses in pharmacology and biomedical research: A consensus framework for design, conduct, and reporting. Adv. Transl. Res..

[198] Alalwan T.A. (2023). Nutraceuticals and their role in promoting musculo-skeletal healthy aging. Ann. Ig. Med. Prev. E Comunita.

[199] Medoro A., Scapagnini G., Davinelli S. (2024). Polyphenol supplementation and sarcopenia: A systematic review and Meta-Analysis of clinical trials. J. Frailty Aging.

[200] Rudrapal M., Rakshit G., Singh R.P., Garse S., Khan J., Chakraborty S. (2024). Dietary polyphenols: Review on chemistry/sources, bioavailability/metabolism, antioxidant effects, and their role in disease management. Antioxidants.

[201] Fekete M., Major D., Feher A., Fazekas-Pongor V., Lehoczki A. (2024). Geroscience and pathology: A new frontier in understanding age-related diseases. Pathol. Oncol. Res..

[202] Kimble R., Jones K., Howatson G. (2023). The effect of dietary anthocyanins on biochemical, physiological, and subjective exercise recovery: A systematic review and meta-analysis. Crit. Rev. Food Sci. Nutr..

[203] Ispoglou T., Wilson O., McCullough D., Aldrich L., Ferentinos P., Lyall G., Stavropoulos-Kalinoglou A., Duckworth L., Brown M.A., Sutton L. (2023). A narrative review of non-pharmacological strategies for managing sarcopenia in older adults with cardiovascular and metabolic diseases. Biology.

[204] Simopoulos A.P. (2022). The Healthiest Diet for You: Scientific Aspects.

[205] Langevin H.M., Weber W., Chen W. (2024). Integrated multicomponent interventions to support healthy aging of the whole person. Aging Cell.

